# Genome-Wide Identification and Functional Characterization of Stress Related Glyoxalase Genes in *Brassica napus* L.

**DOI:** 10.3390/ijms24032130

**Published:** 2023-01-21

**Authors:** Guixin Yan, Meili Zhang, Wenjie Guan, Fugui Zhang, Wenjun Dai, Lili Yuan, Guizhen Gao, Kun Xu, Biyun Chen, Lixia Li, Xiaoming Wu

**Affiliations:** The Key Laboratory of Biology and Genetic Improvement of Oil Crops, The Ministry of Agriculture and Rural Affairs of the PRC, Oil Crops Research Institute of the Chinese Academy of Agricultural Sciences, Wuhan 430062, China

**Keywords:** glyoxalase, *Brassica napus*, genome-wide analysis, expression, stress

## Abstract

Rapeseed (*Brassica napus* L.) is not only one of the most important oil crops in the world, but it is also an important vegetable crop with a high value nutrients and metabolites. However, rapeseed is often severely damaged by adverse stresses, such as low temperature, pathogen infection and so on. Glyoxalase I (GLYI) and glyoxalase II (GLYII) are two enzymes responsible for the detoxification of a cytotoxic metabolite methylglyoxal (MG) into the nontoxic S-D-lactoylglutathione, which plays crucial roles in stress tolerance in plants. Considering the important roles of glyoxalases, the *GLY* gene families have been analyzed in higher plans, such as rice, soybean and Chinese cabbage; however, little is known about the presence, distribution, localizations and expression of glyoxalase genes in rapeseed, a young allotetraploid. In this study, a total of 35 *BnaGLYI* and 30 *BnaGLYII* genes were identified in the *B. napus* genome and were clustered into six and eight subfamilies, respectively. The classification, chromosomal distribution, gene structure and conserved motif were identified or predicted. BnaGLYI and BnaGLYII proteins were mainly localized in chloroplast and cytoplasm. By using publicly available RNA-seq data and a quantitative real-time PCR analysis (qRT-PCR), the expression profiling of these genes of different tissues was demonstrated in different developmental stages as well as under stresses. The results indicated that their expression profiles varied among different tissues. Some members are highly expressed in specific tissues, *BnaGLYI11* and *BnaGLYI27* expressed in flowers and germinating seed. At the same time, the two genes were significantly up-regulated under heat, cold and freezing stresses. Notably, a number of *BnaGLY* genes showed responses to *Plasmodiophora brassicae* infection. Overexpression of *BnGLYI11 gene* in *Arabidopsis thaliana* seedlings confirmed that this gene conferred freezing tolerance. This study provides insight of the *BnaGLYI* and *BnaGLYII* gene families in allotetraploid *B. napus* and their roles in stress resistance, and important information and gene resources for developing stress resistant vegetable and rapeseed oil.

## 1. Background

Rapeseed (*Brassica napus* L.) is one of the most important oil crops in the world. It not only provides high quality edible oil for human beings, but also is a new type of vegetable [1]. Low erucic acid and glucosinolate rapeseed has rich vitamins, fiber and minerals in its stems and leaves and tastes good, which shows great market prospect [2,3]. However, rapeseed is frequently subjected to adverse damage by stresses caused by low temperature, pathogen infection and so on, which not only seriously reduce the yield, but also threatens the health of animals and human beings when it enters the food chain [4]. Thus, exploring and utilizing stress-tolerant genes in rapeseed is of great theoretical and practical significance for vegetable and oilseed production.

Previous reports reveal that the dominant methylglyoxal (MG) concentration in plants can be increased under stress conditions compared with normal conditions [5]. The glyoxalase pathway is the dominant MG scavenging pathways of plants, which includes two enzymes, GLYI and GLYII [6]. GLYI catalyzes the MG conversion into S-D-lactoylglutathione in the presence of reduced glutathione (GSH) [7]. Subsequently, GLYII hydrolyzes S-D-lactoylglutathione into non-toxic D-lactic acid and GSH [8]. The depletion of GSH and the activity of GLYI lead to the toxicity of MG [9]. GLYII is the second rate-limiting enzyme in the system [10]. Lack of GLYII leads to the accumulation of lactoylglutathione [11]. Recently, the third enzyme GLYIII, found to convert MG directly into D-lactose, is far less efficient than the GLYI and GLYII enzymes [11].

In plants, the roles of GLY systems in resistance to biotic and abiotic stresses have been getting more attention recently. The role of *GLY* systems in resisting salt, extreme temperature and heavy metal stresses were reported [12,13,14,15,16]. *GLYI* and *GLYII* can be induced by stresses at transcription and translation levels. For example, in *Brassica juecea, GLYII* expression was significantly up-regulated under zinc stress [17]. The GLYI protein in rice roots at the seedling stage was significantly induced by low temperature stress [18]. The GLYI protein in tomato roots was significantly increased under heavy metal Al stress [19]. Similarly, GLYI and GLYII activities in onion were also induced by low temperature [20]. The level of transcript, protein and specific activity of GLYI was markedly enhanced under water stress [12]. In addition to abiotic stress responses, *GLY* genes are also regulated by biotic stresses. The expression of *GLYI* was induced in *Brassica* after infection by *Sclerotinia sclerotiorum* [21]. In *B. rapa, BraGLYI1*, *BraGLYI6*, *BraGLYI11* and *BraGLYI16* were up-regulated after inoculation of *Plasmodiophora brassicae* in both clubroot-resistant and susceptible lines of *B.rapa*. *BraGLYII13* was more highly induced in the resistant line than the susceptible line at 12 h after inoculation [22]. Overall, the enzyme system is considered as a biomarker of plant stress tolerance [23].

A high concentration of MG can impair plant cells or cell components and can even destroy DNA or cause mutation, resulting in the death of plant cells and tissues [16]. Overexpression or higher activity of glyoxalase enzymes eliminate MG toxicity and confer stress tolerance [23]. For example, overexpression of the *GLYI* gene in tobacco showed better stress tolerance than the normal plant to MG and high salinity [13]. The transgenic mustard overexpressing the *GLYI* gene enabled tolerance to salt, heavy metals and drought stresses [5]. Overexpressing of wheat’s *GLYI* gene also enhanced the tobacco tolerance to ZnCl_2_ [24]. Double *GLY* transgenic tomato plants (*BjGlyI* from *B. juncea* and *PgGlyII* from *Pennisetum glaucum*) showed improved salinity resistance, probably by decreasing the oxidative stress [15]. The transgenic tobacco overexpressing both *GLYI* and *GLYII* genes showed significant resistance to ZnCl_2_ [25]. The overexpression of *Manihot esculenta GLYI-13* enhanced the growth ability of transgenic yeast under iron stress [26]. Therefore, overexpression of the glyoxalase pathway or individual genes harbors the potential to confer tolerance to multiple stresses.

Both *GLYI* and *GLYII* genes were present as multi-gene families. To date, genome-wide identification of the glyoxalase family has been carried out in *Arabidopsis thaliana*, *Oryza sativa*, *Glycine max*, *Medicago truncatula*, *Brassica rapa*, *B. oleracea*, *Vitis vinifera* and *Manihot esculenta. Crantz* [22,26,27,28,29,30,31,32,33]. There are at least 11 *GLYI* homologous genes in *A. thaliana* and rice, 24 *GLYI* members in the soybean genome and 15 in *B. rapa*. According to *GLYII* genes, five *GLYII* genes are in *A. thaliana*, three in rice, twelve *GLYII* members in the soybean genome, and sixteen *GLYII* in *B. rapa* [22,28]. Moreover, some *GLYI* and *GLYII* family genes have been proved to play an important role in response to drought, heavy metals and other stress responses [34,35,36]. Rapeseed (*B. napus*) is a young allotetraploid, which has more gene copied than its diploid progenitor *B. rapa* and *B. oleracea* [37]. In this study, the members of *GLYI* and *GLYII* families were identified from the *B. napus* database by bioinformatics analysis, and their genetic structure, evolutionary relationships, conserved motifs, chromosome localization, expression in different tissues and response to stresses were also studied. The study will provide an important basis for clarifying the evolutionary level and functional differentiation of *GLYI* and *GLYII* family genes in polyploidy crops and lay a theoretical foundation for further understanding the response mechanisms of stress in *Brassica*.

## 2. Results

### 2.1. Identification of GLYI and GLYII Genes in the B. napus Genome

Glyoxalase proteins (GLYI and GLYII) had been identified in Brassicaceae plant *A. thaliana* and *B. rapa.* Thus, *AtGLYI*/*AtGLYII* and *BraGLYI*/*BraGLYII* sequences were used as queries to blast the genome database of *B. napus*, and the sequences with an E-value under 1.0 × 10^−10^ were screened out. Then, proteins that included the glyoxalase domain (PF00903) and the metallo-beta-lactamase domain (PF00753) were classified as BnaGLYI and BnaGLYII proteins, respectively. In this study, 35 *BnaGLYI* and 30 *BnaGLYII* genes were identified in *B. napus*. Table 1 summarizes the gene ID, chromosomal distribution, number of introns, CDS and amino acid length, predicted sub-cellular localization, calculated molecular weight and isoelectric points of the putative *BnaGLYI* and *BnaGLYII*.

The CDS length of *BnaGLYI* members ranged from 324 bp (*BnaGLYI34*) to 3648 bp (*BnaGLYI06*). Accordingly, *BnaGLYI06* encodes for the largest protein with a polypeptide length of 1216 aa and a molecular weight of 134.9 kDa, while *BnaGLYI34* encodes for the smallest protein with a polypeptide length of 108 aa and a molecular weight of 12.2 kDa (Table 1). The isoelectric point (pI) of BnaGLYI proteins vary from 4.78 (BnaGLYI08) to 8.86 (BnaGLYI06). Most of the BnaGLYI members showed acidic pI value, and only four proteins (BnaGLYI02, BnaGLYI05, BnaGLYI06 and BnaGLYI23) showed obvious alkalinity pI value (around 8.0) (Table 1). Moreover, there were also four members (BnaGLYI04, BnaGLYI19, BnaGLYI24 and BnaGLYI31) showing a basic pI value (Table 1). Sub-cellular localization of all identified BnaGLYI proteins were analyzed using three tools (CELLO, Wolf-pSORT and ChloroP). The results predicted that all BnaGLYI, except BnaGLYI15, were localized in cytoplasm. However, the TargetP predicted that most BnaGLYI were localized in the chloroplast, followed by the cytoplasm, nucleus, mitochondrion and cytoskeleton (Table 1).

Similarly, the CDS length of *BnaGLYII* genes also had very wide ranges, from 777 bp (*BnaGLYII09*) to 5745 bp (*BnaGLYII24*). Accordingly, BnaGLYII09 was the smallest protein (259 aa, 28.57 kDa), while *BnaGLYII24* was the largest protein (1915 aa, 212.36 kDa) (Table 2). The pI value of BnaGLYII proteins varied from 5.21 (BnaGLYII29) to 9.04 (BnaGLYII04). Most of the BnaGLYII proteins showed alkalinity with a pI value around or more than 7.0, and only seven BnaGLYII proteins had an acidic pI value, including BnaGLYII09, BnaGLYII12, BnaGLYII13, BnaGLYII15, BnaGLYII17, BnaGLYII29 and BnaGLYII30 (Table 2). Sub-cellular locations of BnaGLYII proteins were different from BnaGLYI proteins. BnaGLYII protein members were localized in different sub-cellular compartments, such as cytoplasm, chloroplast, extracellular matrix, outermembrane, periplasm, nucleus and mitochondrion (Table 2).

### 2.2. Chromosome Localization and Gene Duplication Analysis of BnaGLYI and BnaGLYII

**Figure 1:**
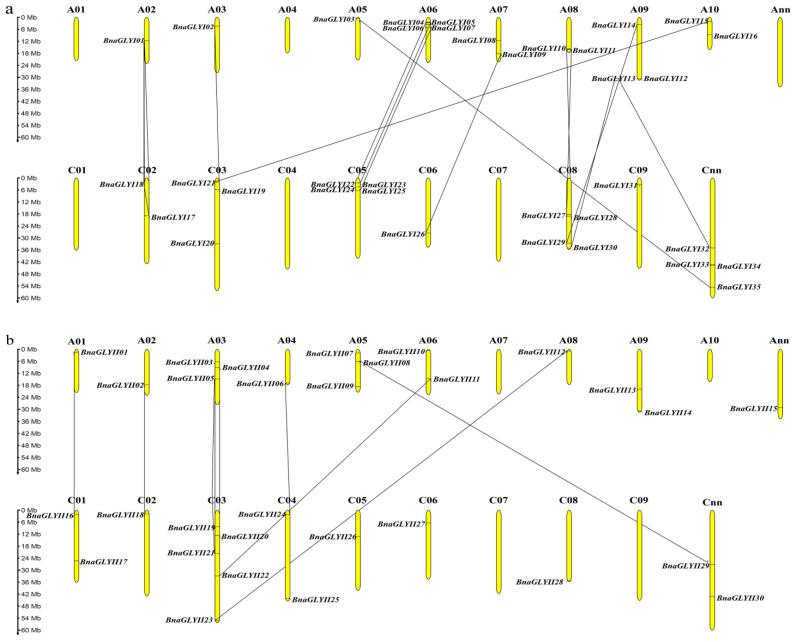
Chromosomal localization of *BnaGLYI* (**a**) and *BnaGLYII* (**b**) genes in the *B. napus* chromosomes. Duplicated genes are connected by black lines.

In order to get a more intuitive view of the distribution of *GLY* genes in *B. napus* chromosomes, the information of their starting and ending position in chromosomes were collected (Table 1), and a chromosome distribution map was drawn on the MG2C (Map Gene2 Chromosome v2) website (Figure 1). For the *BnaGLYI* gene family, there were 31 genes that were unevenly distributed on 14 chromosomes, and no genes were found on chromosomes A01, A04, C01, C04 and C07. Four genes (*BnaGLYI32-35*) were located in the unmapped scaffolds in the Cnn_ random genome (Figure 1a). Chromosomes A06, C05 and C08 contained the maximum number of *BnaGLYI* genes, four genes each, while five chromosomes (A02, A03, A05, C06 and C09) had only one *BnaGLYI* gene (Figure 1a). In total, 16 *BnaGLYI* genes were distributed on the An-subgenome and 19 were from the Cn-subgenome. Similarly, 27 *BnaGLYII* genes were unevenly located in 15 different chromosomes (Figure 1b). There were four chromosomes that contained no *BnaGLYII* genes (A07, A10, C07 and C09) (Figure 1b). Chromosome C03 harbored five *BnaGLYII* genes, which contained the most *BnaGLYI* genes. However, most of chromosomes, like A01, A02, A04, A08, C02, C05, C06 and C08, contained only one *BnaGLYII* gene (Figure 1b). The remaining three genes could not be mapped into chromosomes of the rapeseed genome, which were distributed on Ann and Cnn (Figure 1b). Moreover, BnaGLYII proteins were mainly localized in the chloroplast and cytosol.

Gene replication is the main way of gene family expansion. This study also analyzed the gene replication of the *GLY* gene family in *B. napus*. Among BnaGLYI proteins, there were 15 pairs of duplicated genes. Three genes categorized into one group that formed three gene pairs (*BnaGLYI01*/*17*/*18*). Other gene pairs all included two genes (*BnaGLYI02*/*19*, *BnaGLYI03*/*35*, *BnaGLYI04/22*, *BnaGLYI05/23*, *BnaGLYI07/25*, *BnaGLYI09/26*, *BnaGLYI10/28*, *BnaGLYI11/27*, *BnaGLYI12/32*, *BnaGLYI13/30*, *BnaGLYI14/29* and *BnaGLYI15/21*). Moreover, there were nine gene pairs with over 95% sequence similarity in BnaGLYII proteins. They were *BnaGLYII01/16*, *BnaGLYII02/18*, *BnaGLYII03/19*, *BnaGLYII04/20*, *BnaGLYII05/21*, *BnaGLYII06/24*, *BnaGLYII08/29*, *BnaGLYII11/22* and *BnaGLYII12/23*, respectively (Figure 1b). Such a high level of sequence similarity implied the possibility of segmental duplication of those genes. In addition, there were no tandem duplications found in both the BnaGLYI and BnaGLYII proteins.

### 2.3. Gene Structure and Phylogenetic Analysis of the BnaGLYI and BnaGLYII

**Figure 2:**
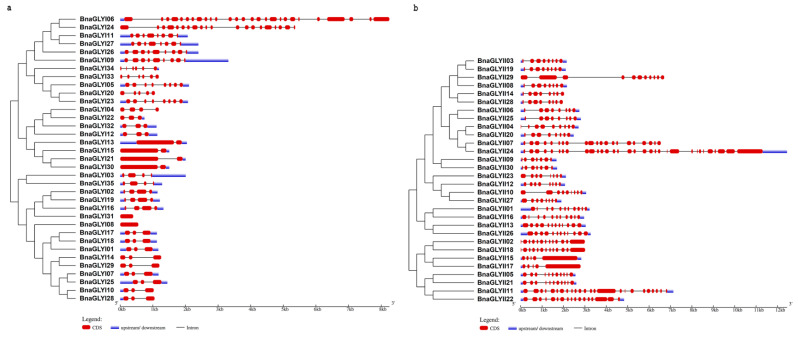
Phylogenetic relationship and gene structure of *BnaGLYI* and *BnaGLYII*. (**a**) BnaGLYI, (**b**) BnaGLYII. An unrooted tree was generated by MEGA 7.0 software (the bootstrap value is 1000) using the full-length amino acid sequences. Exons and untranslated regions (UTR) are indicated by rectangles (red and blue) and introns by lines. CDS and amino acid sequences of *BnaGLYI* and *BnaGLYII* are listed in additional data sheet (Table S5 and S6).

To further investigate the gene structure, the CDS and genomic sequences of the *BnaGLY* genes were aligned. One to twenty-six introns varied from *BnaGLYI* genes, and there were no introns in the *BnaGLYI08* and *BnaGLYI31* (Figure 2b, Table 1)*. BnaGLYI13*, *BnaGLYI15*, *BnaGLYI21* and *BnaGLYI30* only contained one intron. *BnaGLYI06* gene had the largest number of introns (26). Similarly, the *BnaGLYII* genes also contained varied numbers of introns (4–31), e.g., thirty-one introns were identified in *BnaGLYII17*, and four introns were predicted in *BraGLYII24* (Figure 2b, Table 2). In addition, the GLY proteins that clustered in the same group possessed a similar structure (Figure 2).

**Figure 3:**
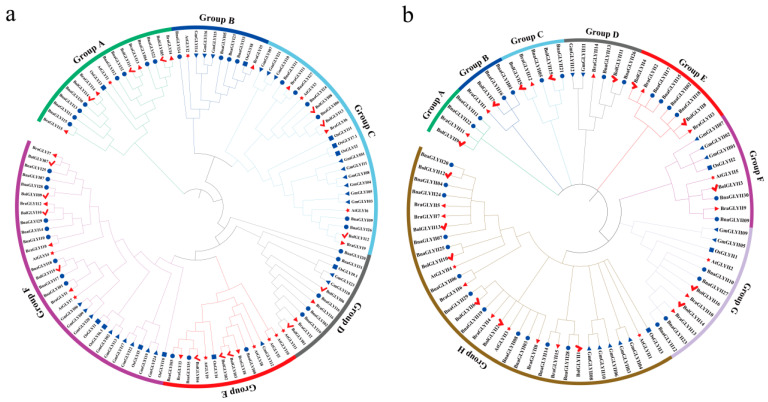
Phylogenetic relationships of BnaGLYΙ (**a**) and BnaGLYII (**b**) from various plant species. A phylogenetic tree was constructed based on the multiple alignment results, using MEGA 7.0 software with the Neighbor-Joining method. Bootstrap support from 1,000 reiterations is indicated above the branches. The 112 GLYI proteins from *B. napus* (35), *B. rapa* (16), *B. oleracea* (15), *Arabidopsis* (11) *G. max* (24) and *O sativa* (11) were used in phylogenetic analysis of BnaGLYΙ (**a**), and the 81 GLYII proteins from *B. napus* (30), *B. rapa* (15), *B. oleracea* (16), *Arabidopsis* (5), *G. max* (24) and *O sativa* (3) were used in phylogenetic analysis of BnaGLYΙI (**b**). Only the first splice variants were considered in the case of multiple members, respectively. “At”, “Bra”, “Bol”, “Gm” and “Os” refer to the proteins in *A. thaliana*, *B. rapa*, *B. oleracea*, *G. max* and *O sativa*. Proteins from *B. napus*, *B. rapa*, *B. oleracea*, *Arabidopsis*, *G. max* and *O sativa* are marked with solid circles, red triangles, check marks, stars, blue triangles and squares, respectively. The sequences used in the analysis are listed in additional data sheet (Table S7).

To understand the evolutionary relationships and functions among the predicted BnaGLY proteins, a phylogenetic tree was drawn with all BnaGLY protein sequences by MEGA7.0. The thirty-five BnaGLY proteins were resolved into six distinguishing groups (Group A–F; Figure 3a). The largest clade Group F, containing thirty-two GLYI members, included nineteen GLYI in Brassicaceae, whereas the smallest group (Group B), containing ten members, included six GLYI in Brassicaceae (Figure 3a). Group A included sixteen members of Brassicaceae, and one protein from rice. Group C included twenty-six proteins, which contained fourteen members of Brassicaceae (six BnaGLYI, three BraGLYI, three BolGLYI and two AtGLYI). Group D and E contained 13 and 14 members, respectively. Generally, BnaGLYI members first clustered with proteins from *B. rapa* and *B. oleracea*, followed by AtGLYIs, forming a small Brassicaceae clade, and subsequently further clustered with GLYI members from rice (Figure 3a).

Similarly, the BnaGLYII proteins formed eight distinct groups (Figure 3b). Two BnaGLYII proteins were clustered in groups A, B, C, D and F, respectively; four BnaGLYII proteins were classified in group E and G, and twelve BraGLYII proteins were in Group H (Figure 3b).

To explore the selective pressure on *BnaGLY* genes, we calculated the non-synonymous/synonymous mutation ratio (Ka/Ks); Ka/Ks > 1 indicates positive selection, Ka/Ks = 1 indicates neutral selection, and Ka/Ks < 1 indicates purifying selection [38]. The Ka/Ks ratio for 10 *BnaGLYI* genes was >1, ranging from 1.0664 (*BnaGLY11*) to 6.3118 (*BnaGLY102*), which indicated that these genes were experienced higher positive selection pressure during the evolutionary history of *B. napus*. The other genes showed Ka/Ks < 1, which experienced higher purifying selection pressure (Table S1). Moreover, most of the *BnaGLYII* genes had a Ka/Ks < 1, and only five *BnaGLYII* genes experienced positive selection pressure (Table S2). A comparison of Ka/Ks ratios among the *BnaGLYI* and *BnaGLYII* genes indicated that the average Ka/Ks ratio of *BnaGLYI* was higher than that of *BnaGLYII* genes. These results suggested that the *BnaGLYI* gene family may have suffered robust purifying selective pressure during evolution.

### 2.4. Conserved Motif and the Enzyme Activity of BnaGLY

**Figure 4:**
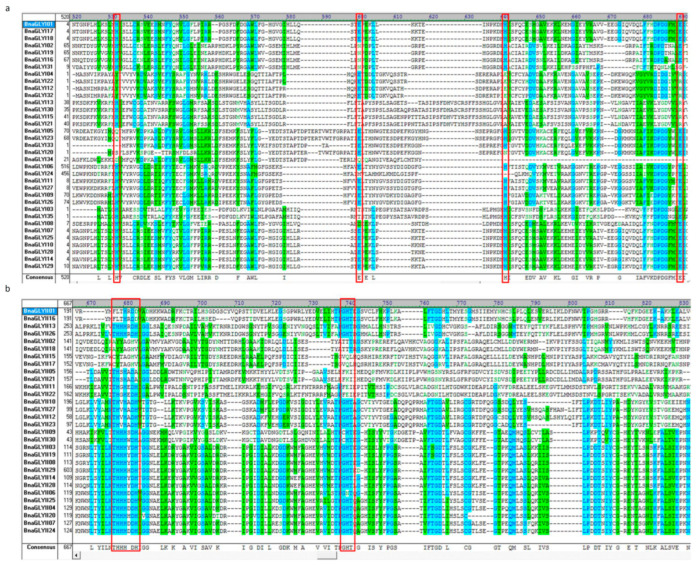
Multiple sequence alignment of GLY domain of all BnaGLYI and BnaGLYII proteins. (**a**) The conserved amino acids (H/QEH/QE) of BnaGLYI were marked by red boxes. (**b**) The red boxes indicate the most conserved metal binding motif (THHHXDH) and active site motif (G/CHT) of BnaGLYII proteins.

Based on the conserved metal binding sites H/QEH/QE of GLYI proteins and multiple alignment results, the expected enzyme activity of the putative BnaGLYI proteins was predicted. Overall, out of a total thirty-five putative BnaGLYI proteins, sixteen have all the four conserved residues and are expected to have functional GLYI enzyme activity (Figure 4 and Table S3). The other 19 BnaGLYI proteins may have variant function. Additionally, GLYII from microorganisms and plants contained the well-conserved metal binding motif (THXHXDH) and active site motif (C/GHT) [31,39], which play an important role in GLYII enzyme activity. Therefore, based on the protein sequence alignment results, the presence of enzymatic activity of the putative BnaGLYII proteins were evaluated (Figure 4, Table S4). Fourteen of thirty putative BnaGLYII proteins possess all the conserved metal binding residues and may have the functional GLYII enzyme activity (Table S4).

To better understand the protein sequence features of BnaGLY, the conserved motifs of each protein were also identified using the MEME Suite 5.1.1 (http://meme-suite.org/tools/meme, (accessed on 28 May 2020)) (Figure S1). We found that most proteins in the same group had similar motifs, and the LOGOs of these protein motifs were obtained by MEME (Figure S2). In BnaGLYI proteins, motif 1 was observed in all the 34 BnaGLYI proteins, except the BnaGLYI20. Motifs 1, 2, 3 and 4 contained the conserved sites of H/QEH/QE. Similarly, motif 1 was detected in most BnaGLYII proteins, except the BnaGLY16 and BnaGLY26. Motifs 1 and 3 contain the metal binding site (THXHXDH) and active site (C/GHT), respectively. Moreover, we also found that most proteins in the same group had similar motifs (Figure S2). However, the conserved domain of the *BnaGLY* protein was divergent (Figure S3a,b). The structural domain is not as conservative as expected.

### 2.5. Tissue Expression Profiles of BnaGLYI and BnaGLYII

To investigate the tissue-specific expression profiles of the *BnaGLY* genes, we selected 20 types of tissues in database (https://biodb.swu.edu.cn/brassica/, (accessed on 15 June 2020)) to analyze, including 10 tissues at the initial flowering stage and 10 tissues at the different development stages of the podding. The expression levels of the *BnaGLY* genes showed diverse expression patterns (Figure S3). Six *BnaGLYI* members (*BnaGLYI09*, *BnaGLYI11*, *BnaGLYI16*, *BnaGLYI26*, *BnaGLYI27* and *BnaGLYI35*) were highly expressed in most of the tissues. Their average fragments per kilo base of exons per million fragments mapped (FPKM) values were over twenty-five, and the value in a single tissue was at least over three. In contrast, the expression levels of *BnaGLYI22*, *BnaGLYI29*, *BnaGLYI32* and *BnaGLYI34* were not detected in most tissues. They only showed faint expression in one to three tissues, e.g., the expression of *BnaGLYI32* was only detected in silique pericarps (SP) at 24 and 27 days after flowering (DAF). The expression of *BnaGLYI14* was not detected in all the 10 tissues (Figure S4a). In *BnaGLYII* genes, *BnaGLYII28* was only weakly expressed in SP at 27 DAF, while the FPKM values of *BnaGLYII18* and *BnaGLYII29* were less than one. The other *BnaGLYII* genes were constitutive expression. However, some genes were expressed at higher levels in specific tissues, e.g., *BnaGLYII19* was highly expressed in anther (Figure S4b). These results indicated that most *BnaGLY* genes were expressed at different levels in different tissues.

**Figure 5:**
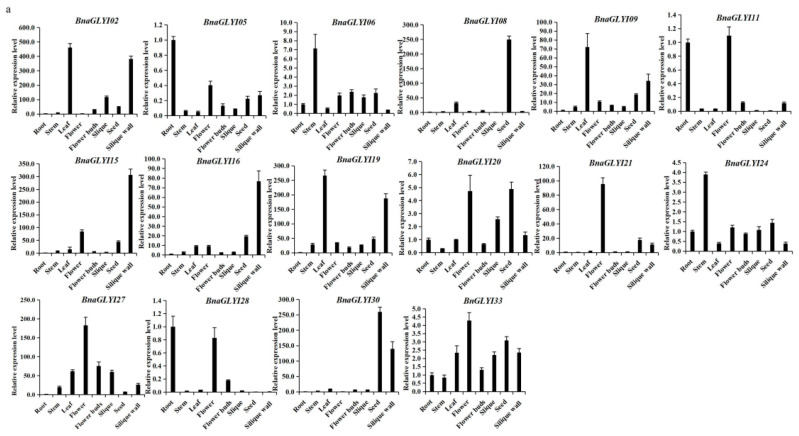

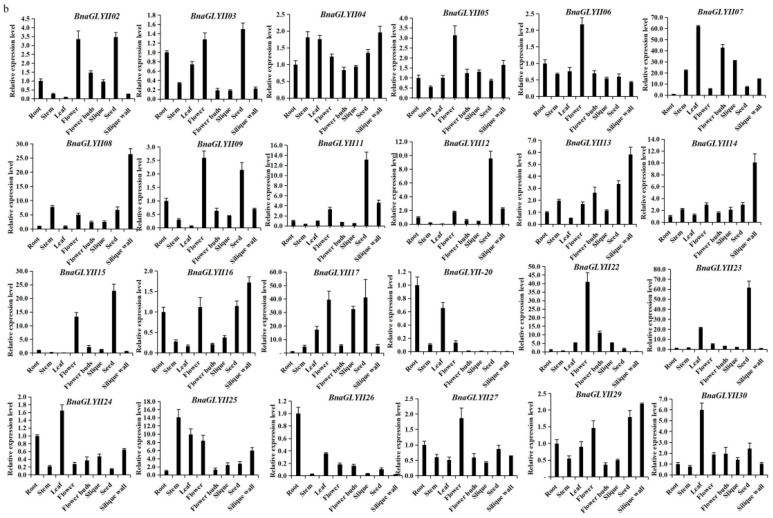
Relative expressions of the *BnaGLYI* (**a**) and *BnaGLYII* (**b**) genes in different tissues of *B. napus* confirmed by qRT-PCR. The normalized relative quantity in the root was set as “1”.

The expression levels of some *BnaGLY* genes were verified in eight different tissues and organs (including root, stem, leaf, flower, flower bud, 25DAF seed, silique and silique wall by qRT-PCR (Figure 5). A total of 19 *BnaGLYI* genes and 33 *BnaGLYII* genes were selected after verifying the specificity for each primer pair. According to the data, the expression level of some *BnaGLYI* and *BnaGLYII* genes was consistent with the results of database; for example, the expression level of most *BnaGLYI* was not detected, such as *BnaGLYI29* and *BnaGLYII28* (Figure S5 and Table S8), whereas most *BnaGLYII* showed constitutive expression. Some genes expressed highly in specific tissues; for example, *BnaGLYI02*, *BnaGLYI09* and *BnaGLYI19* showed relatively high expression levels in the leaf and silique wall, *BnaGLYII12* and *BnaGLYII15* were highly expressed in seeds and *BnaGLYII05* was highly expressed in flowers. However, the expression level of several genes was inconsistent with the GEO data; for example, *BnaGLYI11* and *BnaGLYI27* showed particularly high expression levels in the flowers (Figure 5a), and *BnaGLYII20* and *BnaGLYII26* were strongly expressed in the root (Figure 5b). Moreover, qRT-PCR results indicated that *BnaGLYII07* and *BnaGLYII24* were much more highly expressed in the leaf, not in the stem as shown in Figure 5b.

The identified paralogous pairs of *BnaGLYI* and *BnaGLYII* genes revealed similar expression patterns, such as BnaGLYI02/19. However, some of the paralogous gene pairs showed a high level of expression divergence in different tissues. For example, *BnaGLYII06* showed high level expression in flowers, while its paralogous *BnaGLYII24* showed high level expression in leaf. The diverse expression patterns suggested that these members might play diverse functions.

Moreover, the expression levels of *BnaGLY* in developing seeds at two, four, six and eight weeks after pollination (WAP) were analyzed using the gene expression omnibus (GEO) database (GSE77637) (Figure S6). The expression of *BnaGLYI11* and *BnaGLYI27* genes was high in the four stages, and the FPKM values were all over 2000. *BnaGLYI01*, *BnaGLYI04*, and *BnaGLYI32* showed high expression level at eight WAP. *BnaGLYI10*, *BnaGLYI14*, *BnaGLYI28, BnaGLYI29* and *BnaGLYI34* showed low expression levels in all the seed development stages (Figure S6a). In the case of *BnaGLYII*, the expression of *BnaGLYII09*, *BnaGLYII14* and *BnaGLYII28* was up-regulated at eight WAP. The expressions of *BnaGLYII03*, *BnaGLYII06*, *BnaGLYII24*, *BnaGLYII13*, *BnaGLYII25*, *BnaGLYII19*, *BnaGLYII20* and *BnaGLYII26* were gradually decreased in the four development stages (Figure S6b).

### 2.6. Expression of BnaGLY in B. napus Seed Germination

To dissect the influence of *GLY* pathways in the seed germination of *B. napus*, the expression level of *GLY* genes was analyzed using the data in GEO (GSE13723). The expression level of *BnaGLYI* in winter oilseed rape (WOSR) accessions with different germination rates (high, medium and low; C129, C033 and C032, respectively) at different germination times showed similar expression patterns, e.g., the expression of *BnaGLYI11* and *BnaGLYI27* were significantly up-regulated at 72 h after imbibition (hai) in all the three accessions, and the expressions of *BnaGLYI04*, *BnaGLYI07*, *BnaGLYI17*, *BnaGLYI21* and *BnaGLYI25* obviously decreased at 36 hai and 72 hai. Meanwhile, *BnaGLYI06*, *BnaGLYI10*, *BnaGLYI14*, *BnaGLYI24*, *BnaGLYI28*, *BnaGLYI29* and *BnaGLYI34* showed weak expression in all the accessions at the four germination stages (Figure S7a). In contrast, the average expression level of *BnaGLYII* genes was much higher than that of *BnaGLYI. BnaGLYII5*, *BnaGLYII11*, *BnaGLYII21* and *BnaGLYII22* showed significant up-regulation at 36 hai and 72 hai in all the three accessions, while *BnaGLYII1* was only up-regulated at 72 hai in the three accessions. *BnaGLYII9* showed high expression at early stages of germination; however, it was sharply decreased at 36 hai, and then was significantly up-regulated at 72 hai. *BnaGLYII3* and *BnaGLYII19* were up-regulated at the three germination time points in the three accessions. *BnaGLYII17* and *BnaGLYII25* showed very low expression levels in accession C033 from 0 to 72 hai (Figure S7b). *BnaGLY* showed diverse expression patterns, though some genes were clustered within the same group.

### 2.7. The Response of the BnaGLYI and BnaGLYII on Pathogen Infection

**Figure 6:**
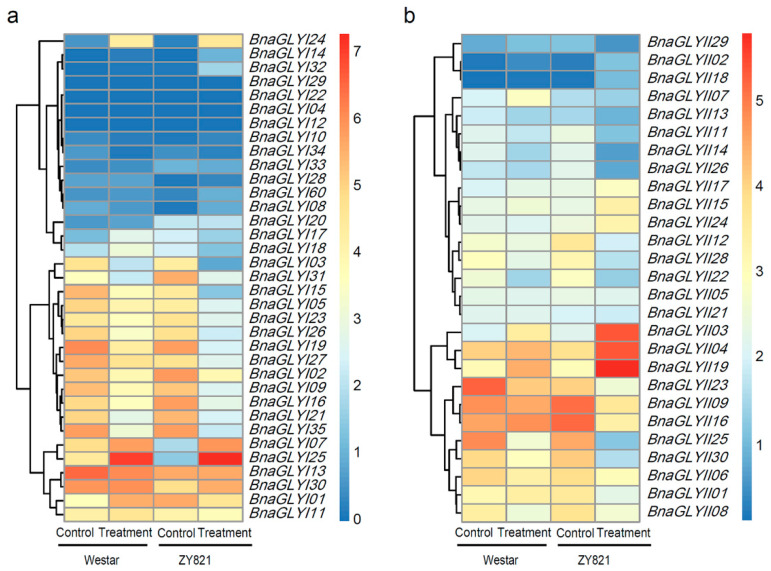
Expression profiles of the *BnaGLY* genes in response to *P. brassicae* infection. Relative expression data of available *BnaGLYI* (**a**) and *BnaGLYII* (**b**) genes under *P. brassicae* infection were obtained from BrassicaEDB database (https://biodb.swu.edu.cn/brassica/, accessed on 15 June 2020). The expression level of the inoculated leaves in susceptible (Westar) and tolerant (ZY821) genotypes were analyzed. Note: CK: Control condition; TR: Treatment under *P. brassicae* infection.

The RNA-seq data sets available in GEO (GSE81545) were used for analysis of the *BnaGLY* gene expression in response to the fungal pathogen [40]. In the *BnaGLYI* gene, the expression levels of *BnaGLYI07*, *BnaGLYI24* and *BnaGLYI25* were significantly up-regulated after *S. sclerotiorum* pathogen infection in the resistant cultivar (ZY821) and sensitive cultivar (Westar). *BnaGLYI02*, *BnaGLYI19, BnaGLYI31* and *BnaGLYI35* were significantly down-regulated after pathogen infection in both cultivars, while the expression of *BnaGLYI08* and *BnaGLYI10* was down–regulated in sensitive cultivar; however, their expression was up–regulated in resistant cultivar. Most interestingly, the *BnaGLYI17* and *BnaGLYI18* genes were up-regulated in the susceptible cultivar; however, they were down-regulated in the resistant cultivar (Figure 6a).

In contrast to the *BnaGLYI* genes, most *BnaGLYII* genes showed significantly decreased expression levels after pathogen infection, e.g., *BnaGLYII06*, *BnaGLYII08*, *BnaGLYII14*, *BnaGLYII23*, *BnaGLYII25*, *BnaGLYII28* and *BnaGLYII30* were significantly down-regulated in both cultivars. Only *BnaGLYII02*, *BnaGLYII03*, *BnaGLYII18* and *BnaGLYII19* were significantly induced in both cultivars after being infected by pathogen (Figure 6b). Moreover, *BnaGLYII16* was up-regulated in the susceptible cultivars and down-regulated in the resistant cultivars.

### 2.8. Expression of BnaGLY in Response to Abiotic Stresses

**Figure 7:**
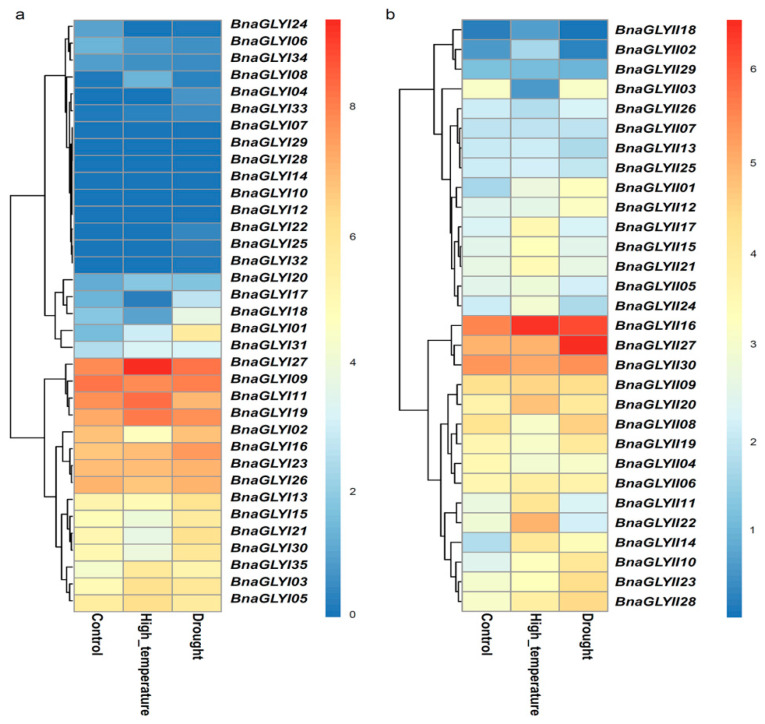
Expression profiles of the *BnaGLY* genes in response to heat and drought stresses. Relative expression data of available *BnaGLYI* (**a**) and *BnaGLYII* (**b**) genes under heat (40 °C, 3 h) and drought (withdrawing water, 3 days) treatment were obtained from NCBI GEO database (GSE156029, https://www.ncbi.nlm.nih.gov/geo/query/acc.cgi?acc=GSE156029, (accessed on 16 December 2022)).

We also investigated the response of *BnaGLY* genes to drought and heat stresses at expression level (Figure 7, Table S9). The expression levels of 12 *BnaGLYI* genes (*BnaGLYI01, BnaGLYI03, BnaGLYI05, BnaGLYI08, BnaGLYI11, BnaGLYI19, BnaGLYI20, BnaGLYI27, BnaGLYI31, BnaGLYI33* and *BnaGLYI35*) were up-regulated under heat stress, while the expression of 12 *BnaGLYI* genes (*BnaGLYI02, BnaGLYI06, BnaGLYI09, BnaGLYI13, BnaGLYI15, BnaGLYI17, BnaGLYI18, BnaGLYI21, BnaGLYI24, BnaGLYI26, BnaGLYI30* and *BnaGLYI34*) were down-regulated under heat stress. Meanwhile, twenty *BnaGLYI* were highly expressed under heat stress, and five *BnaGLYI* were lowly expressed under drought stress. Ten *BnaGLYI* genes (*BnaGLYI01, BnaGLYI03, BnaGLYI08, BnaGLYI16, BnaGLYI19, BnaGLYI20, BnaGLYI27, BnaGLYI31, BnaGLYI33* and *BnaGLYI35)*, whose expression levels were up-regulated under heat stress, were also highly expressed under drought stress, while four *BnaGLYI* genes (*BnaGLYI06*, *BnaGLYI09*, *BnaGLYI24* and *BnaGLYI34*) that were down-regulated under heat stress were equally low expressed under drought stress. At the same time, the expression levels of *BnaGLYI11* increased under heat stress but decreased under drought stress, while six *BnaGLYI* genes (*BnaGLYI13, BnaGLYI15*, *BnaGLYI17*, *BnaGLYI18, BnaGLYI21* and *BnaGLYI30*) decreased under heat stress but increased under drought stress (Figure 7a).

In the case of *BnaGLYII*, twenty and fourteen *BnaGLYII* genes were induced under heat and drought stresses, respectively, while six and ten *BnaGLYII* genes were lowly expressed under heat and drought stresses, respectively. Among these genes, ten genes (*BnaGLYII01, BnaGLYII06, BnaGLYII09, BnaGLYII10, BnaGLYII14, BnaGLYII16, BnaGLYII20, BnaGLYII23* and *BnaGLYII28*) were up-regulated and two genes (*BnaGLYII04* and *BnaGLYII29*) were down-regulated under both stresses (Figure 7b). At the same time, the expression levels of six *BnaGLYII* genes (*BnaGLYII02*, *BnaGLYII05, BnaGLYII11*, *BnaGLYII13*, *BnaGLYII18* and *BnaGLYII24*) increased under heat stress but decreased under drought stress, while four *BnaGLYII* genes (*BnaGLYII08, BnaGLYII19, BnaGLYII26* and *BnaGLYII30)* decreased under heat stress but increased under drought stress (Figure 7b).

**Figure 8:**
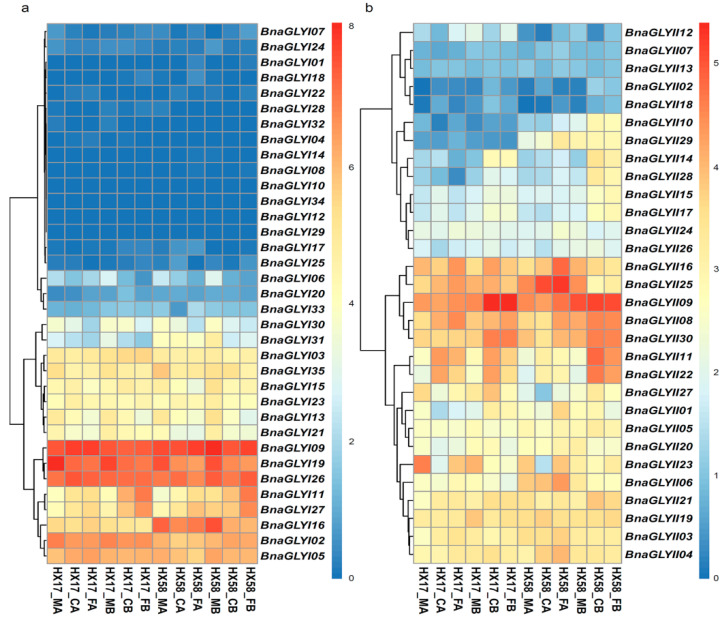
Expression of *BnaGLY* genes under different low-temperature treatments. (**a**) *BnaGLYI* genes; (**b**) *BnaGLYI* genes. The expression levels of each gene (log_2_ (FPKM values + 1)) in each sample is indicated by different color rectangles. The transcripts of each gene in leaves treated with chilling and freezing, with or without cold acclimation, were determined using the data from GEO (GSE129220). HX17 and HX58 were two early maturing semi-winter rapeseed varieties. This shows the expression level of *BnGLY* genes in the two *B. napus* varieties with different cold tolerances under cold accumulations at chilling (CA) and freezing (FA) temperature and cold shocks at the same temperature (CB and FB) condition. MA and MB were the control with and without cold accumulations, respectively.

To detect the response of *BnaGLY* genes to low temperature stress, the FPKM value of each gene (Table S10), treated with different cold tolerance under cold accumulations at chilling (CA) and freezing (FA) temperature and cold shocks at the same temperature (CB and FB) condition in two *B. napus* varieties, were determined using the data from GEO (GSE129220: https://www.ncbi.nlm.nih.gov/geo/query/acc.cgi?acc=GSE129220, (accessed on 16 December 2022)). A total of nine, twelve, ten and thirteen *BnaGLYI* genes were up-regulated under CA, FA, CB and FB treatments compared with their controls in *B. napus* variety HX17, respectively. Among these up-regulated genes, there were one (*BnaGLYI11*), two (*BnaGLYI27* and *BnaGLYI11*) and one (*BnaGLYI11*) genes significantly up-regulated under CA, FA and CB treatment (Figure 8a and Table S9). There were nine, eight, four and seven *BnaGLYI* genes up-regulated under CA, FA, CB and FB treatments, and two (*BnaGLYI27* and *BnaGLYI11*), one (*BnaGLYI11*) and three *BnaGLYI* (*BnaGLYI07, BnaGLYI11* and *BnaGLYI27*) genes significantly up-regulated under CA, FA, and FB treatments in *B. napus* variety HX58.

In the case of *BnaGLYII*, 13, 21, 17 and 17 *BnaGLYII* genes were induced in HX17, while 16, 25, 17 and 18 *BnaGLYII* genes were induced under CA, FA, CB and FB treatments HX58, respectively (Figure 8b and Table S10). Among these genes, three (*BnaGLYII11*, *BnaGLYII22* and *BnaGLYII02*), four (*BnaGLYII02, BnaGLYII11, BnaGLYII14* and *BnaGLYII22*) and four (*BnaGLYII01, BnaGLYII09, BnaGLYII14* and *BnaGLYII30*) were up-regulated significantly under CA, CB and FB in HX17. Three (*BnaGLYII04*, *BnaGLYII11* and *BnaGLYII22*), eight (*BnaGLYII01, BnaGLYII04, BnaGLYII16, BnaGLYII18, BnaGLYII22, BnaGLYII24, BnaGLYII25* and *BnaGLYII29*), eight (*BnaGLYII02, BnaGLYII10, BnaGLYII11, BnaGLYII14, BnaGLYII17, BnaGLYII18, BnaGLYII22* and *BnaGLYII28*) and nine (*BnaGLYII02, BnaGLYII10, BnaGLYII11, BnaGLYII14 BnaGLYII15, BnaGLYII17, BnaGLYII18, BnaGLYII22* and *BnaGLYII28*) *BnaGLYII* were up-regulated significantly under CA, FA, CB and FB in HX58.

### 2.9. Overexpression of BnGLYI Confer Enhanced Arabidopsis Seedlings Freezing Tolerance

**Figure 9:**
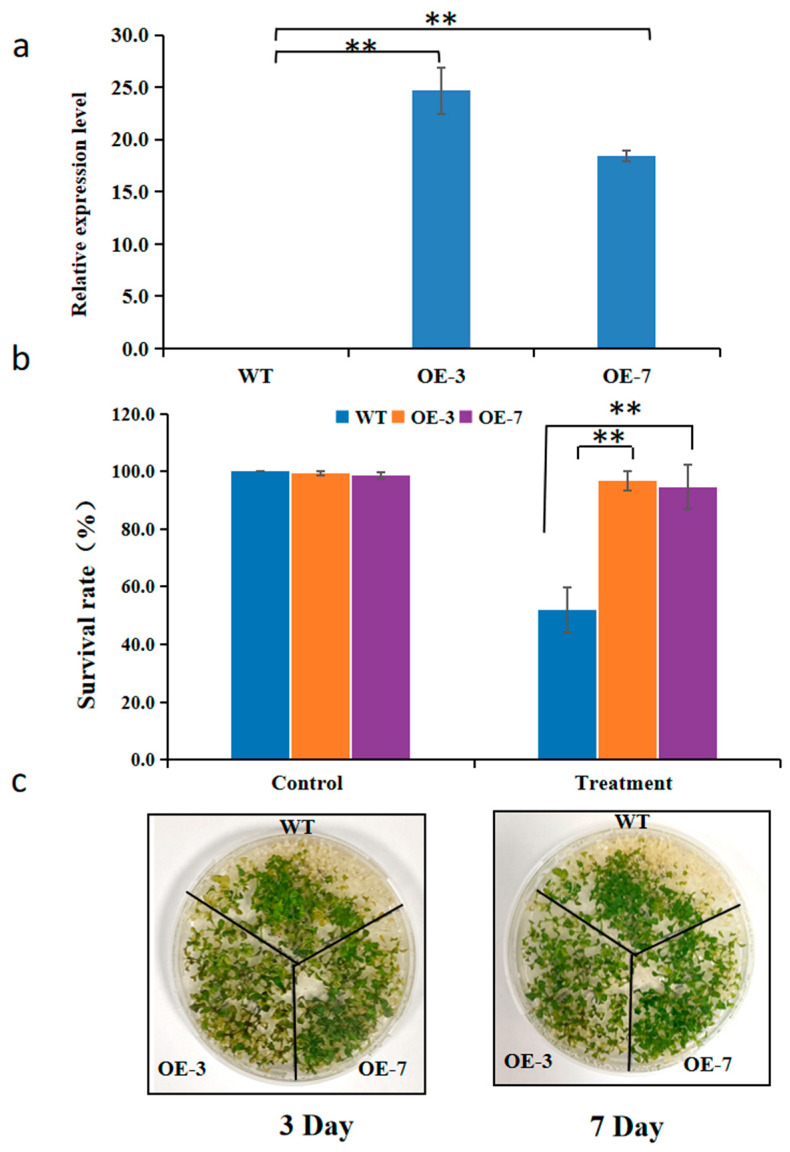
The function of the *BnaGLYI11* gene in response to cold stress. (**a**) The expression level of *BnaGLYI11* in overexpression *A. thaliana* lines OE-3 and OE-7 in 7-day-old seedling. (**b**) Survival rate after −10 °C for cold stress treatment. Two-week-old seedlings grown on MS medium (22 °C with 8 h of light daily) were treated at −10 °C for 210 min after 4 °C for three days. After cold treatment, the seedlings were incubated at 4 °C in the dark for 12 h and then transferred to normal grown conditions. The survival rates of the seedlings were scored visually after 7 days. WT: wild-type (Col-0), OE: *BnaGLYI11* gene overexpression lines. (**c**) phenotype of wild-type (Col-0) and transgenic *BnaGLYI11* gene after −10 °C treatment. Asterisks represent the statistical significance between the means for each treatment. ** *p* < 0.001. Vertical bars in the graph represent mean ± SD.

The above results revealed that *BnaGLYI11* was significantly induced under both chilling and freezing stresses regardless of cold acclimation, which indicated that the two genes play important roles in cold tolerance in *B. napus.* To elucidate the function of *BnaGLY11* gene, we examined the freezing tolerance of *BnaGLYI11*–overexpressing transgenic *Arabidopsis thaliana* seedlings (*BnaGLYI11*-OE). *BnaGLYI11* was indeed overexpressed in these transgenic plants (Figure 9a). After the freezing treatment, the *BnGLYI11*-OE plants showed higher survival rates than the wild-type plants under freezing conditions. The results indicated that the plants over expressing the *BnGLYI11* gene possessed increased capacities to tolerate freezing temperatures (Figure 9b,c).

## 3. Discussions

Abiotic and biotic stresses severely affect the overall growth of plants and reduce their total productivity. MG is a cytotoxic metabolite, which is produced more under abiotic and biotic stresses [23]. Excessive accumulation of MG results in the disruption of the antioxidant defense system, biomembrane structures and cellular functions, including the peroxidation of lipids, the oxidation of protein and fatty acids and other metabolic dysfunctions [23,41]. The glyoxalase pathway, as the main MG detoxification system, could protect DNA and protein by converting MG into D-lactate and improve plant adaptation to multiple stresses. Many studies have revealed that the glyoxalase-overexpressing transgenic plants restrict MG accumulation and confer stress tolerance [23]. Therefore, genome-wide analysis of the glyoxalase enzymes has been carried out previously in rice, *Arabidopsis*, soybean, *B. rapa,* grape and *M. truncatula* based on their conserved metal ion and substrate binding sites [22,27,28,29,31,42]. However, these families have not been studied in *B. napus,* the important oil and vegetable crop. In this study, we identified 35 *BnaGLYI* and 30 *BnaGLYII* in *B. napus* genome. *B. napus* (AACC) originates from natural crossing of *B. rapa* (AA) and *B. oleracea* (CC) [43]. Sixteen *BraGLYI* and fourteen *BraGLYII* were located in the AA subgenome, while fifteen and thirteen *BolGLYI* and *BolGLYII* were located in the CC subgenome. The number of *BnaGLYI* gene number was much higher than the ancestral species plants *B. rapa* [22] and *B. oleracea* [33] (total 31 *GLYI*). The GLY families display varied sequences, which might lead to diversity in their biochemical functions. Furthermore, phylogenetic relations, gene structures, protein motifs, Ks/Ka and the expression pattern of *BnaGLY* gene families were systematically investigated in the study.

According to the recent GLYI screening criteria, not only metal binding sites but also GSH binding sites, active sites and dimer interfaces should be considered [44]. Meanwhile, fourteen conserved amino acids of GLYIIs were important for substrate binding, metal binding and catalytic activity [45]. Using these criteria, only three *A. thaliana* genes (*AtGLYI2, AtGLYI3* and *AtGLYI6)* and two rice genes (*OsGLYI8* and *OsGLYI11.2*) were functional and contained all the binding sites. In this study, different BnaGLYs subfamily proteins shared the different type of conserved domains, which suggested their functional diversity. Analysis of all BnaGLYI proteins revealed that 16 BnaGLYI possessed conserved metal binding sites (H/QEH/QE) and may have expected enzyme activity of the putative BnaGLYI proteins along with the catalytic domain (PF00903). In addition, fourteen of thirty putative BnaGLYII proteins contained a metal binding motif (THHHXDH) and an active site motif (C/GHT), which were similar to *Escherichia coli*, *Saccharomyces cerevisiae*, *Salmonella typhimurium*, *L. infantum*, *Homo sapiens* and higher plants (*A. thaliana*, *B. juncea*, *O. sativa*) [39].

Previous reports indicated that genes with less introns showed rapid gene activation and timely responses to various stresses [46]. In our study, *BnaGLYI* and *BnaGLYII* genes showed significantly varied gene length and exon-intron structure. However, the genes with less or no introns did not show higher expression levels as reported [46,47], e.g., *BnaGLYI08* and *BnaGLYI31* containing no introns showed very low level of expression, which did not show significant expression responses to the stress conditions. Meanwhile, we found that some genes expressed higher in specific tissues; for example, *BnaGLYI02*, *BnaGLYI09* and *BnaGLYI19* were highly expressed in the leaf, whereas *BnaGLYII12* and *BnaGLYII15* were highly expressed in seeds and *BnaGLYII05* was highly expressed in flowers. These results indicate that most *BnaGLY* genes were expressed at different levels in different tissues. In addition, the expression of several *BnaGLYI* and *BnaGLYII* genes detected using qRT-PCR was different from the published database. This was understandable, as there were different sampling periods and growth conditions.

There is a firm link between GLY enzymes and stress tolerance in plants. The expression level of GLYI and GLYII can be induced under various stress treatments in different plants [34]. The transcripts of *GLYI* in tomatoes were up-regulated under salinity stress and phytohormonal and osmotic stimulation [34]. In pumpkin seedlings, the transcription level of *GLYI* was induced by salinity, heavy metal, white light and MG treatments [48]. Therefore, the expression patterns of the *BnaGLYI* and *BnaGLYII* genes were first analyzed using publicly available expression data in the study. The *BnaGLY* did not show any significant effect on winter oilseed rape germination rates, because the genes showed a similar expression pattern in accessions with different germination rates during germination. In a recent study, the glyoxalase enzyme of rice not only increased tolerance abiotic stresses (salinity, drought and extreme temperatures) but also reduced damage from biotic stresses (*Rhizoctonia solani*) [49]. In grapes, most glyoxalase genes had two periods of high expression after downy mildew inoculation [27]. In our study, the induced genes after infection may have a response on the *S. sclerotiorum* pathogen, e.g., *BnaGLYI07*, *BnaGLYI24*, *BnaGLYI25*, *BnaGLYII02*, *BnaGLYII03*, *BnaGLYII18* and *BnaGLYII19* genes, whose expressions were highly up-regulated in both cultivars. Furthermore, the genes that were only up–regulated in resistant cultivar may have related to the disease resistances of plants, such as *BnaGLYI08* and *BnaGLYI10*. In addition, the genes up-regulated in the susceptible cultivar and down-regulated in the resistant cultivar require further study, such as the *BnaGLYI17* and *BnaGLYI18*. However, whether glyoxalase proteins participate in *S. Sclerotiorum* pathogen response require further investigations.

In this study, we also uncovered that *BnaGLYI* and *BnaGLYII* genes are highly responsive to drought, heat and cold stresses, which implied these genes contribute to multiple stress responses in *B. napus*. For example, *BnaGLYI27* was highly up-regulated under both heat drought stresses, and *BnaGLYII22* was significantly induced under both chilling and freezing stresses regardless of cold acclimation. Most importantly, *BnaGLYI11* was significantly up-regulated under all temperature stresses (heat, chilling and freezing stresses, regardless of cold acclimation). Considering rapeseed oil suffers cold stress during vegetative stage, the rapeseed varieties with low cold tolerance have higher risk of freeze injury in cold winter and spring. Hence, it is vital to understand the cold-induced molecular responses in rapeseed. Therefore, *BnaGLYI11* was overexpressed in *A. thaliana* and the transgenic *A. thaliana* with the *BnaGLYI11* gene confers freezing tolerance. Previously, we found *BnaGLYI* (BnaA06g04580D, *BnaGLYI05*) transgenic yeast cells enhanced their tolerance to extreme temperature stress [30]. Similarly, overexpression of *AtGLYI2*, *AtGLYI3* and *AtGLYI6* in *E. coli* provides multi-stress heat tolerance [44]. BnaGLYI11 shared approximately 96% identity with ATGLYI3, and BnaGLYΙ05 showed 98% identity with ATGLYI2 (Table S11). The alignment results indicated that BnaGLY proteins shared a high sequence similarity, which may show significant function similarity. However, the freezing-tolerant BnaGLYΙ11 showed low similarity to BnaGLYΙ05 (35%), which indicates that the cold tolerance function may be due to the conserved function domains. Further investigations should explore the mechanism of the response of the glyoxalase pathway to stress tolerance in plants to generate more stress-tolerant varieties using molecular approaches.

## 4. Conclusions

This study provides a systematic knowledge of *BnaGLY* gene family in *B. napus*. A total of 35 *BnaGLYI* and 30 *BnaGLYII* genes were identified. The genes in the same subfamily showed similar structure and motif composition. Phylogenetic comparison and synteny analysis of *BnGLY* genes provide valuable clues for their evolutionary characteristics. Moreover, the gene expression pattern and their responses to seed gemination rate, *S. Sclerotiorum* pathogen heat and drought and temperature stresses were also determined. The transgenic *A. thaliana* with the temperature stresses responding to *BnaGLYI-11* confers cold tolerance. These results provide an important foundation for further understanding the biological functions of *BnaGLY* genes and their utilization in rapeseed breeding.

## 5. Methods

### 5.1. Plant Materials

The *B. napus* cultivar Zhongshuang11 used for edible oil and vegetable plants was obtained from the National Mid-term Gene Bank of Oil Crops Research Institute, Chinese Academy of Agriculture Sciences. It was planted in a growth chamber at 20 ± 2 °C with 12 h light and 12 h dark. The roots were sampled from 3-leaf stage seedlings. Fresh flower buds were obtained, and stems, leaves, siliques and seeds were sampled 25 days after flowering (DAF). Three biological replicates of each tested tissue were collected from three different individuals. All the samples were immediately frozen in liquid nitrogen and stored at −80 °C until RNA isolation. Wild-type *Arabidopsis* (Columbia ecotype) and overexpression transgenic lines were planted in a growth room with a constant temperature and light cycle (16 h light/8 h dark, 22 ± 1 °C, humidity 60%).

### 5.2. Identification of the GLYI and GLYII Genes Family in B. napus

Firstly, AtGLYI and AtGLYII protein sequences were acquired from the Arabidopsis Information Resource-TAIR (http://www.arabidopsis.org, (accessed on 12 May 2020)). The genes and proteins of *B. napus* were downloaded from the website of the *B. napus* Genome Browser (http://www.genoscope.cns.fr/brassicana pus/, (accessed on 18 May 2020)) databases. Secondly, Arabidopsis GLY protein sequences were used as query to perform the Blast P against *B. napus* genome. Then the protein sequences with the e-value under 1.0 × 10^−10^ were confirmed again by the Pfam protein family database, according to the particular domains (PF00903) in GLYI proteins and the metallo-beta-lactamase domain (PF00753) in the GLYII proteins. All identified putative GLY proteins were designated as *BnaGLYI01* to *BnaGLYI35* and *BnaGLYII01* to *BnaGLYII30* according to their location in the chromosome. Localization of proteins were predicted using the CELLO v.2.5 website (http://cello.life.nctu.edu.tw/, accessed on 20 May 2020) [50] and the WOLF PSORT website (https://www.genscript.com/wolf-psort.html, accessed on 20 May 2020) [51]. Chloroplast localization was confirmed by ChloroP (http://www.cbs.dtu.dk/services/ChloroP/, accessed on 20 May 2020) [52]. The isoelectric points (pI) and molecular weights (Mw) were calculated using the ExPASy proteomics server database (http://www.expasy.org/tools/, (accessed on 26 May 2020))

### 5.3. Chromosomal Location, Duplication and Ka (Non-Synonymous Mutation Rate)/Ks (Synonymous Mutation Rate) Analysis

The location information of *GLY* genes on chromosomes were acquired from the website of the *B. napus* Genome Browser. Mapping of these *GLY* genes was performed using the online website MG2C (http://mg2c.iask.in/mg2c_v2.0/, (accessed on 10 August 2020)). Gene duplication was defined according to the criteria described in previous studies [53,54]: the aligned region of two sequences covers over 70% of the longer sequence and the similarity of the aligned region is over 95%. In addition, the Ka and Ks values of the repeating gene pairs were calculated using DnaSp software, and then evolutionary rate and the type of selection pressure on the gene were determined based on the Ka/Ks ratio [38].

### 5.4. Phylogenetic Analysis, Gene Structure and Conserved Motif

The Cluster X program was used to perform multiple sequence alignments with default parameters [55]. Phylogenetic trees were subsequently constructed by the MEGA 6.0 software using Neighbor-Joining (NJ) [56]. Bootstrap analysis was conducted with 1000 replications. The iTOL website (https://itol.embl.de/, (16 August 2020)) was used to better visualize the phylogenetic tree [57].

The exon/intron of *GLY* genes were inferred through comparison of genomic sequences and CDS sequences in the gene structure display server (http://gsds.cbi.pku.edu.cn/index.php) [58].

The MEME online tool (http://meme-suite.org/, (accessed on 28 May 2020)) [59] was employed to identify conserved motifs in *BnaGLY* genes with the following parameters: distribution of motifs, the optimum width of motif, 6–50; the maximum number of motif, 5; the number of repetitions, any. Only motifs with an e-value < 1.0 × 10^−10^ were retained for further analysis.

The conserved domains were retrieved, using NCBI Conserved Domain Database (https://www.ncbi.nlm.nih.gov/Structure/cdd/wrpsb.cgi, (accessed on 23 December 2022)) and Visualized NCBI CDD pattern of TB-tools (https://github.com/CJ-Chen/TBtools, (accessed on 27 December 2022)) [60].

### 5.5. Expression Analysis of BnaGLY Genes at Different Developmental Stages and Under Stress Treatments

The temporal and spatial expression patterns of *BnaGLY* were analyzed using the RNA-seq data online (https://biodb.swu.edu.cn/brassica/, (accessed on 15 June 2020)), including roots, stems, leaves, flowers, seeds and silique tissues and germination, bolting, initial flowering and podding stages. Stem-1 was sampled at the bolting stage. Stem-2, anther, filament, pedicel, inflorescence tip, mature leaf, young leaf, petal and carpel root were sampled at the initial flowering stage. The embryo, inner integument, outer integument, seed, silique pericarp, embryo and seed coat were sampled at podding stage. All the materials were planted in the field.

The publicly available data of the gene expression omnibus (GEO) database (GSE13723) was used to reveal the probable function of *BnaGLY* genes in the germination of winter oilseed rape with different germination rate [61]. The expression of all *BnaGLYI* genes in response to *Sclerotinia* infection was analyzed using the reported RNA-seq data (GSE81545). The inoculated leaves in both susceptible (Westar) and tolerant (ZY821) genotypes of *B. napus* were analyzed [40]. The RNA-seq data of a 35-day-old leaf with treatment of heat (40 °C, 3 h) and drought (withdrawing water, 3 days) (GSE156029) was used to analysis the response of *BnaGLY* genes to heat and drought stresses. The transcriptome data of *B. napus* under cold (4 °C, 12 h) and freezing (−4 °C, 12 h) (GSE129220) temperatures was downloaded from the NCBI database [62].

The data was used to generate a heatmap using the Heat map Illustrator (HemI, http://hemi.biocuckoo.org/down.php, (accessed on 15 October 2020)) package [45].

### 5.6. Plasmid Construction and Plant Transformation

The coding sequence of *BnaA08g25110D* was amplified by PCR and cloned into PBI121s [63] to generate the over-expression plasmids 35S::BnaGLYI, 35S::BnaGLYI, and 35S::BnaGLYI, respectively. Primers containing restriction enzyme sites for *Xbal* Ⅰ and *Kpn* Ⅰ were used for PCR as follows: BnaGLYI11-F: 5’ AGCTTTCGCGAGCTCGGTACCACAGAAAACTCTCAAAGCCC 3’, BnaGLYI11-R: 5’ TGCCTGCAGGTCGACTCTAGAAACACAGAGAAACACGACAC 3’. The constructs used were confirmed by PCR and sequencing.

All the plasmids were transformed into *Agrobacterium tumefaciens* strain GV3101, and the positive clones identified by sequencing were introduced into 5-week-old Arabidopsis plants (Columbia ecotype) using the floral dip method. Positives were selected on MS plates containing 50 mg L^−1^ Kanamycin. The positive transformants was detected using a forward primer designed to the CaMV 35S sequence (35s up-F: 5’ ATTGATGTGAACATGGTGGAG 3’) and a reverse primer of the gene expression (BnaGLYI11-RT-R: 5’ TTGTCTACCAGGACTGTTTTCC 3’). T1 seeds of PCR-positive transformants were harvested and grown to generation, and T3 generation was used for phenotype identification and gene expression.

### 5.7. RNA Isolation and Transcript Analysis

The gene expression of the definitive positive plants was analyzed by qRT-PCR. The total RNA from WT and Transgenetic plants was isolated using a polysaccharide and polyphenol total RNA isolation kit (BioTeke, RP3201, Wuxi, China). The cDNA was synthesized using a synthesis kit (EasyScript® First-Strand cDNA Synthesis SuperMix, TransGen Biotech, Beijing, China), and the qRT-PCR and data analysis were carried out as descried by Li et al. [64]. Each sample was quantified in triplicate with three biological replicates. The specific primers (BnaGLYI11-RTF: 5’ AGCTGACCTATAACTACGGCG 3’, BnaGLYI11-RTR: 5’ TTGTCTACCAGGACTGTTTTCC3’) designed are used for the detection.

### 5.8. Freezing Tolerance Assay

The freezing tolerance assay was performed as previously described [65], with some modifications. Briefly, seeds were grown 14 days on MS medium at 22 °C with 8 h of light daily. Before cold treatment, the 2-week-old seedlings were plated to 4 °C for three days, and then subjected to 210 min at −10 °C for cold stress treatment. After cold treatment, the seedlings were incubated at 4 °C in the dark for 12 h and then transferred to light at 22 °C. The survival rates of the seedlings were scored visually after 7 days.

## Figures and Tables

**Table 1:** Detail information of *BnaGLYI* genes identified in *Brassica napus* L. genomes.

Gene Symbol	Locus Identifier	Location	Gene Start (bp)	Gene Stop (bp)	Strand	No. of Introns	CDS Length (bp)	PP Length (aa)	MW (kDa)	pI	Localization
*BnaGLYI01*	BnaA02g19970D	A02	12337272	12338438	−	2	504	168	18,818.68	5.81	Cy ^a^; Nu ^b^
*BnaGLYI02*	BnaA03g10440D	A03	4700661	4701804	+	3	582	194	21,785.91	8.43	Cy ^a^; Mt ^b^
*BnaGLYI03*	BnaA05g35240D	A05	670044	672049	−	3	414	138	15,252.22	5.46	Cy ^a^; Nu ^b^
*BnaGLYI04*	BnaA06g04170D	A06	2547123	2548306	−	3	516	172	19,274.7	7.77	Cy ^a^; Ch ^b^
*BnaGLYI05*	BnaA06g04580D	A06	2708014	2710119	+	7	714	238	26,635.17	8.33	Cy ^a^; Om ^a^; Ch ^b,c^
*BnaGLYI06*	BnaA06g07360D	A06	3915659	3923909	+	26	3648	1216	134,984.62	8.86	Cy ^a,b^
*BnaGLYI07*	BnaA06g10060D	A06	5342612	5343786	+	2	525	175	19,778.79	5.68	Cy ^a^; Ch ^b^
*BnaGLYI08*	BnaA07g13890D	A07	12263440	12263994	+	0	555	185	20,887.37	4.78	Cy ^a,b^
*BnaGLYI09*	BnaA07g26290D	A07	19358162	19361477	−	8	1026	342	37,894.44	6.48	Cy ^a^; Ch ^b,c^
*BnaGLYI10*	BnaA08g23870D	A08	16843513	16844540	−	2	525	175	19,846.96	5.88	Cy ^a^; Ch ^b^
*BnaGLYI11*	BnaA08g25110D	A08	17357805	17359871	−	7	852	284	31,872.41	5.26	Cy ^a,b^
*BnaGLYI12*	BnaA09g49270D	A09	32814199	32815336	+	2	414	138	15,394.22	5.84	Cy ^a^; Ch ^b^
*BnaGLYI13*	BnaA09g49870D	A09	33093200	33095241	+	1	1323	441	48,072.12	5.72	Cy ^a^;Om ^a^;Cysk ^b^
*BnaGLYI14*	BnaA09g56790D	A09	3889968	3891223	+	2	504	168	18,943.89	5.86	Cy ^a^; Ch ^b^
*BnaGLYI15*	BnaA10g04310D	A10	2272334	2273834	−	1	1338	446	48,973.02	5.45	Om ^a^; Cy ^b^
*BnaGLYI16*	BnaA10g11070D	A10	9346288	9347612	−	3	588	196	21,959	6.71	Cy ^a^; Mt ^b^
*BnaGLYI17*	BnaC02g23290D	C02	20281012	20282133	−	2	504	168	18,762.53	5.66	Cy ^a^; Nu ^b^
*BnaGLYI18*	BnaC02g46640D	C02	2394713	2395834	+	2	504	168	18,804.61	5.66	Cy ^a^; Nu ^b^
*BnaGLYI19*	BnaC03g13130D	C03	6313042	6314254	+	3	582	194	21,712.86	7.77	Cy ^a^; Ch ^b^
*BnaGLYI20*	BnaC03g51010D	C03	35479070	35480133	−	4	453	151	16,992.13	5.25	Cy ^a^; Nu ^b^
*BnaGLYI21*	BnaC05g04530D	C03	2231485	2233489	−	1	1338	446	48,857.97	5.65	Cy ^a,b^; Om ^a^
*BnaGLYI22*	BnaC05g05340D	C05	2609787	2610529	−	2	417	139	15,596.41	5.94	Cy ^a,b^
*BnaGLYI23*	BnaC05g05770D	C05	2848240	2850316	+	7	708	236	26,438.95	8.74	Cy ^a^; Pe ^a^;Ch ^b,c^
*BnaGLYI24*	BnaC05g08770D	C05	4659651	4665014	+	19	2175	725	80,672.51	7.49	Cy ^a,b^; Om ^a^
*BnaGLYI25*	BnaC05g11680D	C05	6800739	6802181	+	2	525	175	19,733.75	5.68	Cy ^a^; Ch ^b^
*BnaGLYI26*	BnaC06g28360D	C06	29576211	29578606	−	8	1038	346	38,368.97	6.19	Cy ^a^;Ch ^b,c^
*BnaGLYI27*	BnaC08g15100D	C08	19658835	19661229	+	7	852	284	31,814.27	5.26	Cy ^a,b^
*BnaGLYI28*	BnaC08g16660D	C08	20576966	20578018	+	2	525	175	19,821.86	5.89	Cy ^a^; Ch ^b^
*BnaGLYI29*	BnaC08g38920D	C08	34930459	34931660	−	2	522	174	19,568.62	5.86	Cy ^a^; Mt ^b^
*BnaGLYI30*	BnaC08g44820D	C08	37849659	37851160	−	1	1323	441	48,162.21	5.46	Cy ^a^; Cysk ^b^
*BnaGLYI31*	BnaC09g53670D	C09	3644531	3644929	+	0	399	133	15,196.72	7.77	Cy ^a,b^
*BnaGLYI32*	BnaCnng38880D	Cnn	37521585	37522696	−	2	414	138	15,502.36	6.2	Cy ^a^; Ch ^b^
*BnaGLYI33*	BnaCnng47290D	Cnn	46764251	46765432	−	5	432	144	16,180.32	5.39	Cy ^a,b^
*BnaGLYI34*	BnaCnng47280D	Cnn	46758036	46759221	−	5	324	108	12,264.1	5.01	Cy ^a^; Nu ^b^
*BnaGLYI35*	BnaCnng59150D	Cnn	58862580	58863862	−	3	414	138	15,253.16	5.45	Cy ^a^; Nu ^b^

Abbreviations: CDS: coding DNA sequence, PP: polypeptide length, MW: molecular weight, PI: isoelectric point, bp: base pair, aa: amino acid, kDa: kilodalton, Ch: chloroplast, Cy: cytosol, Mt: mitochondria, Nu: nucleus, Pe: periplasm, Ou: outermembrane, Ec: extracellular matrix. ^a^ Localization prediction by pSORT (http://www.genscript.com/wolf-psort.html, (accessed on 20 May 2020)). ^b^ Localization prediction by TargetP 1.1 Server (http://www.cbs.dtu.dk/services/TargetP/, (accessed on 20 May 2020)). ^c^ Chloroplast localization signal confirmed by ChloroP (http://www.cbs.dtu.dk/services/ChloroP/, (accessed on 20 May 2020)).

**Table 2:** Basic characteristics of *BnaGLYII* genes identified in *Brassica napus* L. genomes.

Gene Symbol	Locus Identifier	Location	Gene Start (bp)	Gene Stop (bp)	Strand	No. of Introns	CDS Length (bp)	PP Length (aa)	MW (kDa)	pI	Localization
*BnaGLYII01*	BnaA01g03370D	A01	1597636	1600850	−	10	1056	352	39,338.98	7.93	Cy ^a^; Ch ^b,c^
*BnaGLYII02*	BnaA02g25890D	A02	18962115	18965111	+	12	1848	616	68,076.64	6.62	Cy ^a,b^
*BnaGLYII03*	BnaA03g14380D	A03	6611723	6613870	+	7	987	329	36,085.29	8.81	Cy ^a^; Ch ^b,c^
*BnaGLYII04*	BnaA03g20230D	A03	9624333	9627034	−	8	1026	342	37,496.84	9.04	Ec ^a^; Ch ^b,c^
*BnaGLYII05*	BnaA03g33050D	A03	15994860	15997418	+	10	1074	358	39,988.96	7.17	Cy ^a^; Ch ^b,c^
*BnaGLYII06*	BnaA04g25070D	A04	18183840	18186578	−	7	993	331	36,397.62	8.82	Ec ^a^; Ch ^b,c^
*BnaGLYII07*	BnaA05g03320D	A05	1854330	1860877	−	20	2868	956	106,401.82	8.51	Ou ^a^; Ch ^b,c^
*BnaGLYII08*	BnaA05g11390D	A05	6376663	6378817	−	7	981	327	35,883.92	8.07	Pe ^a^; Ch ^b,c^
*BnaGLYII09*	BnaA05g28080D	A05	20057585	20059255	+	6	777	259	28,689.47	6.1	Pe ^a^; Cy ^b^
*BnaGLYII10*	BnaA06g00850D	A06	603532	606586	+	8	1167	389	43,530.78	7.74	Cy ^a^; Pe^a^; Nu ^b^
*BnaGLYII11*	BnaA06g22690D	A06	15848234	15855367	+	23	3450	1150	126,907.01	9.02	Cy ^a^; Ch^b,c^
*BnaGLYII12*	BnaA08g01070D	A08	806559	808627	+	7	786	262	28,574.49	6.19	Pe ^a^; Nu ^b^
*BnaGLYII13*	BnaA09g28480D	A09	21351908	21354940	−	11	1566	522	57,465.81	6.19	Cy ^a,b^
*BnaGLYII14*	BnaA09g50050D	A09	33176404	33178430	+	7	969	323	35,263.31	7.16	Cy ^a^; Ch ^b,c^
*BnaGLYII15*	BnaAnng27360D	Ann	31283476	31286304	−	4	2085	695	77,448.79	5.81	Cy ^a^; Nu ^b^
*BnaGLYII16*	BnaC01g04650D	C01	2443496	2446457	−	10	1059	353	39,822.41	6.96	Cy ^a^; Ch ^b,c^
*BnaGLYII17*	BnaC01g28980D	C01	27137154	27139952	−	4	2076	692	77,157.53	5.81	Cy ^a^; Mt ^b^
*BnaGLYII18*	BnaC02g47740D	C02	3827265	3830275	+	12	1848	616	68,049.53	6.43	Cy ^a,b^
*BnaGLYII19*	BnaC03g17440D	C03	8920776	8922877	+	7	975	325	35,687.84	8.85	Cy ^a^; Mt ^b^; Ch ^c^
*BnaGLYII20*	BnaC03g24200D	C03	13557749	13560223	−	7	981	327	35,840.97	8.85	Ec ^a^; Pe ^a^; Ch ^b, c^
*BnaGLYII21*	BnaC03g38140D	C03	23395036	23397633	+	10	1077	359	39,993.97	7.14	Cy ^a^; Ch ^b,c^
*BnaGLYII22*	BnaC03g50930D	C03	35336691	35341520	−	16	2682	894	98,725.64	8.7	Cy ^a^; Ch ^b,c^
*BnaGLYII23*	BnaC03g69760D	C03	59489758	59491865	−	7	870	290	31,928.74	8.57	Cy ^a^; Ch ^b,c^
*BnaGLYII24*	BnaC04g02920D	C04	2066379	2078825	−	31	5745	1915	212,357.58	8.24	Ou ^a^; Ch ^b,c^
*BnaGLYII25*	BnaC04g48930D	C04	47330538	47333344	−	7	996	332	36,386.59	8.7	Ec ^a^; Ch ^b,c^
*BnaGLYII26*	BnaC05g20710D	C05	14195563	14198830	+	11	1566	522	57,524.85	6.47	Cy ^a,b^
*BnaGLYII27*	BnaC06g06420D	C06	6895047	6896947	−	7	909	303	33,196.01	7.21	Pe ^a^; Ch ^b,c^
*BnaGLYII28*	BnaC08g44630D	C08	37747241	37749209	−	7	984	328	35,631.65	6.83	Pe ^a^; Ch ^b,c^
*BnaGLYII29*	BnaCnng30690D	Cnn	29167588	29174293	+	10	2448	816	93,535.33	5.21	Cy ^a,b^
*BnaGLYII30*	BnaCnng46950D	Cnn	46379687	46381389	−	6	777	259	28,653.35	5.72	Pe ^a^; Ch ^b,c^

Abbreviations: CDS: coding DNA sequence, PP: polypeptide length, MW: molecular weight, PI: isoelectric point, bp: base pair, aa: amino acid, kDa: kilodalton, Ch: chloroplast, Cy: cytosol, Mt: mitochondria, Nu: nucleus, Pe: periplasm, Ou: outermembrane, Ec: extracellular matrix. ^a^ Localization prediction by pSORT (http://www.genscript.com/wolf-psort.html, (accessed on 20 May 2020)). ^b^ Localization prediction by TargetP 1.1 Server (http://www.cbs.dtu.dk/services/TargetP/, (accessed on 20 May 2020)). ^c^ Chloroplast localization signal confirmed by ChloroP ((http://www.cbs.dtu.dk/services/ChloroP/, accessed on 20 May 2020)).

## Data Availability

Not applicable.

## Supplementary Materials

The following supporting information can be downloaded at: https://www.mdpi.com/article/10.3390/ijms24032130/s1.

## Abbreviations

aa: amino acid; bp: base pair; Br: *Brassica rapa*; BRAD: *Brassica* database; CDS: Coding DNA sequence; Ch: chloroplast; Cy: cytosol; Ec: extracellular matrix; FPKM: Fragments Per Kilo base of exon sper Million fragments mapped; GLYI: glyoxalase I; GLYII: glyoxalase II; GSH: Glutathione (reduced); hai: Hours after inoculation; Kb: Kilo base pair; MG: Methylglyoxal; Mt: mitochondria; MW: molecular weight; NJ: Neighbor-joining; Nu: nucleus; GEO: gene expression omnibus; ROS: Reactive oxygen species; Os: *Oryza sativa*; Ou: outermembrane, Pe: periplasm; *Pg*: *Pennisetum glaucum*; PI: isoelectric point; PP: polypeptide length; ROS: Reactive oxygen species; hai: hour after imbibition
