# Supplementary material for: Genome-Wide Identification and Functional Characterization of Stress Related Glyoxalase Genes in Brassica napus L
Source: Int J Mol Sci. 2023 Jan 21;24(3):2130. doi: 10.3390/ijms24032130 (PMC9916435; doi:10.3390/ijms24032130)
Supplement: Supplementary file 1 [file ijms-24-02130-s001.zip › ijms-2090795-supplementary/Supplementary Materials.pdf]

Supplementary Materials

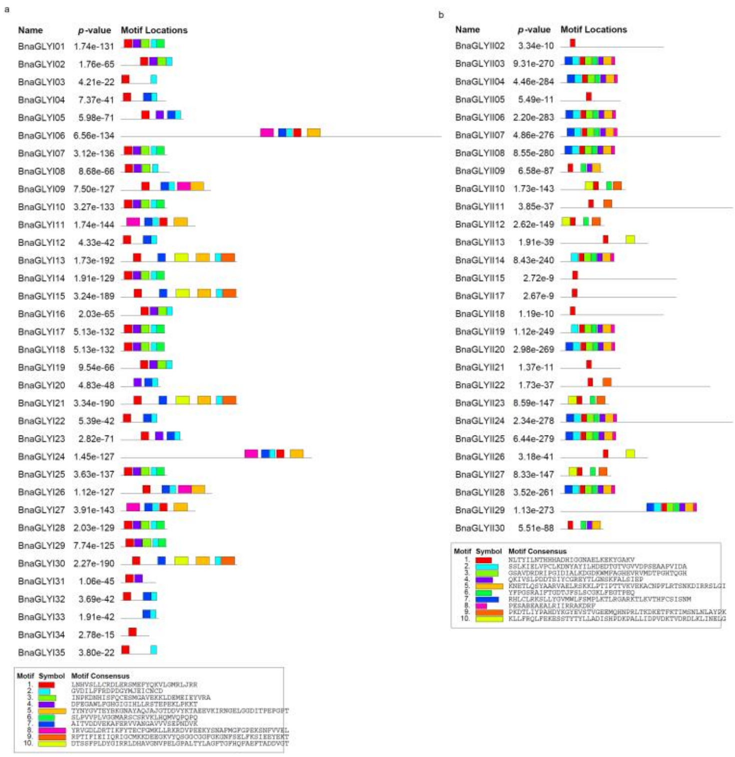

Figure S1. The conserved Motif of BnaGLY proteins (a) and BnaGLYII (b) proteins.

a

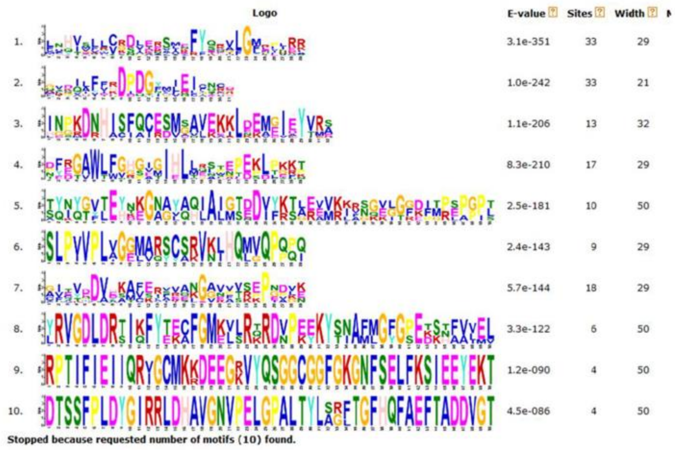

b

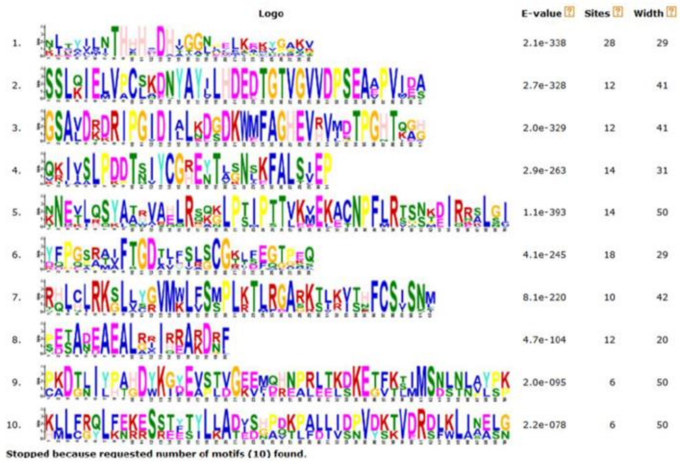

Figure S2. Sequence logo view of the consensus BnaGLYI (a) and BnaGLYII (b) protein motifs in *B. napus*. The height of a letter indicates its relative frequency at the given position.

a

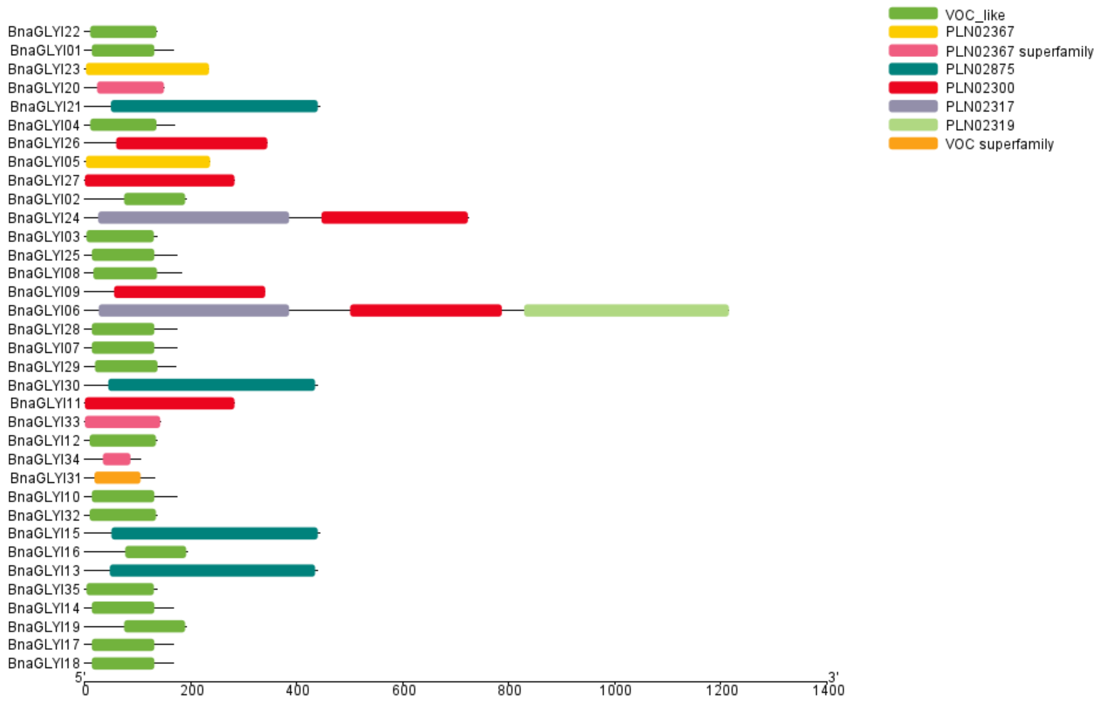

b

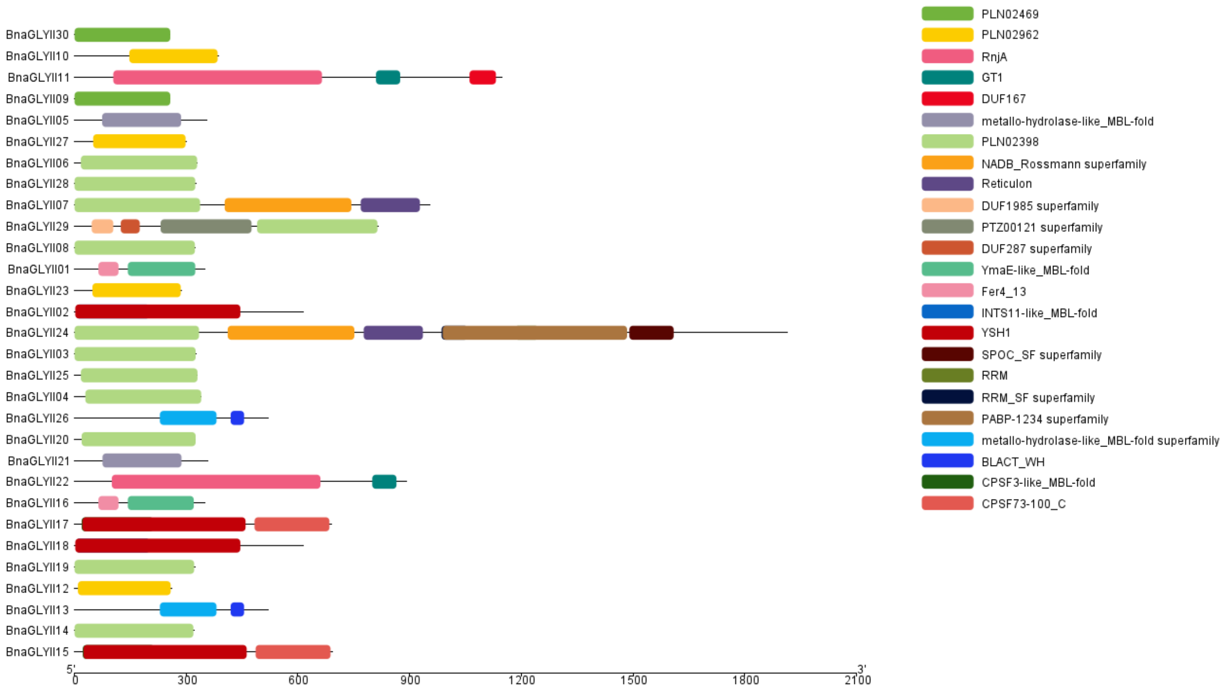

Figure S3. Conserved domain structure of BnaGLYI (a) and **BnaGLYII** (b) protein

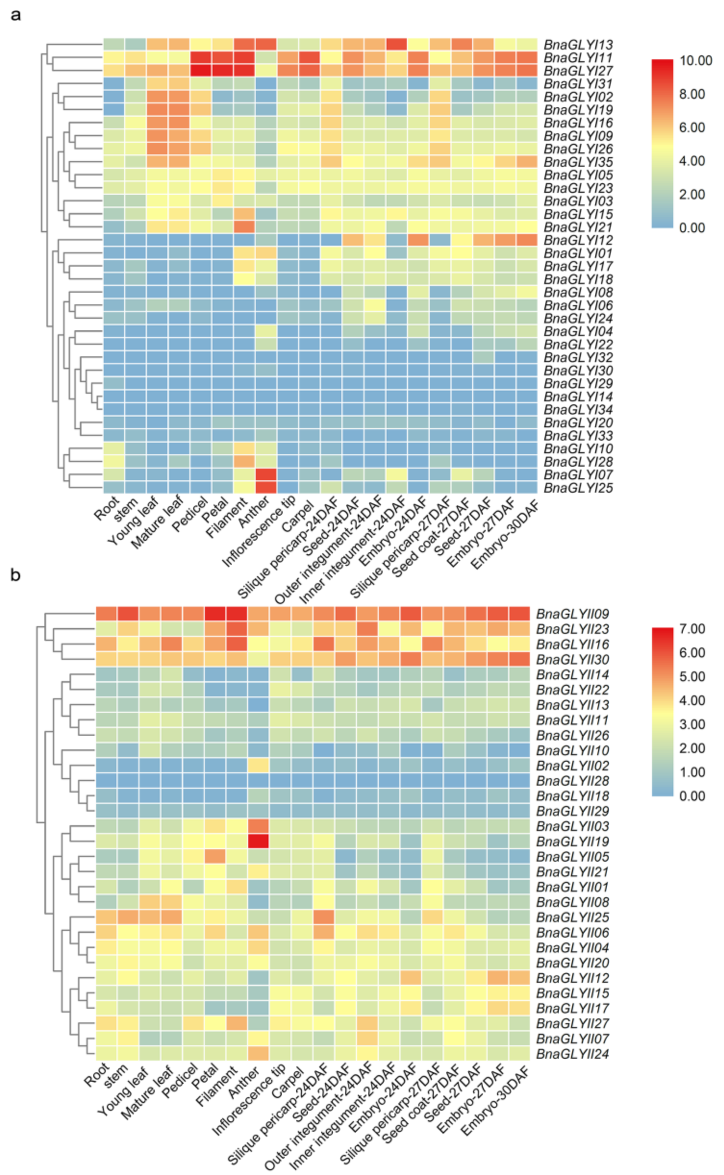

Figure S4. Expression patterns of the *BnaGLY* genes across various tissues and organs. (a) The expression profiles of the *BnaGLYI* genes; (b) The expression profiles of the *BnaGLYII* genes.

The expression level of the *BnaGLY* genes root , stem , young leaf, mature leaf, pedicel, petal, filament, anther, inflorescence tip, and carpel, silique pericarp-24 DAF, seed-24 DAF, outer integument-24 DAF, inner integument-24 DAF, embryo-24 DAF, silique pericarp-27 DAF, seed coat-27 DAF, seed-27 DAF, embryo-27 DAF and embryo-30 DAF. The RNA-seq data was obtained from BrassicaEDB database (https://biodb.swu.edu.cn/brassica/). The Heatmap was generated by Heatmap Illustrator (HemI, http://hemi.biocuckoo.org/down.php) package. The color bar at the upper right side of the figure represents Log$_2$ (FPKM + 1), with blue representing little or no expression.

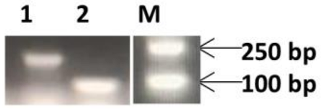

1: PCR product amplified with *BnGLYI29* qRT-PCR primer-pairs using genomic DNA as template (238 bp)
2: PCR product amplified with *BnGLYIi28* qRT-PCR primer-pairs using genomic DNA as template (86 bp)
M: Marker

Figure S5. PCR product amplified with *BnaGLY* genomic DNA as templates to verify qRT-PCR results of the expression genes

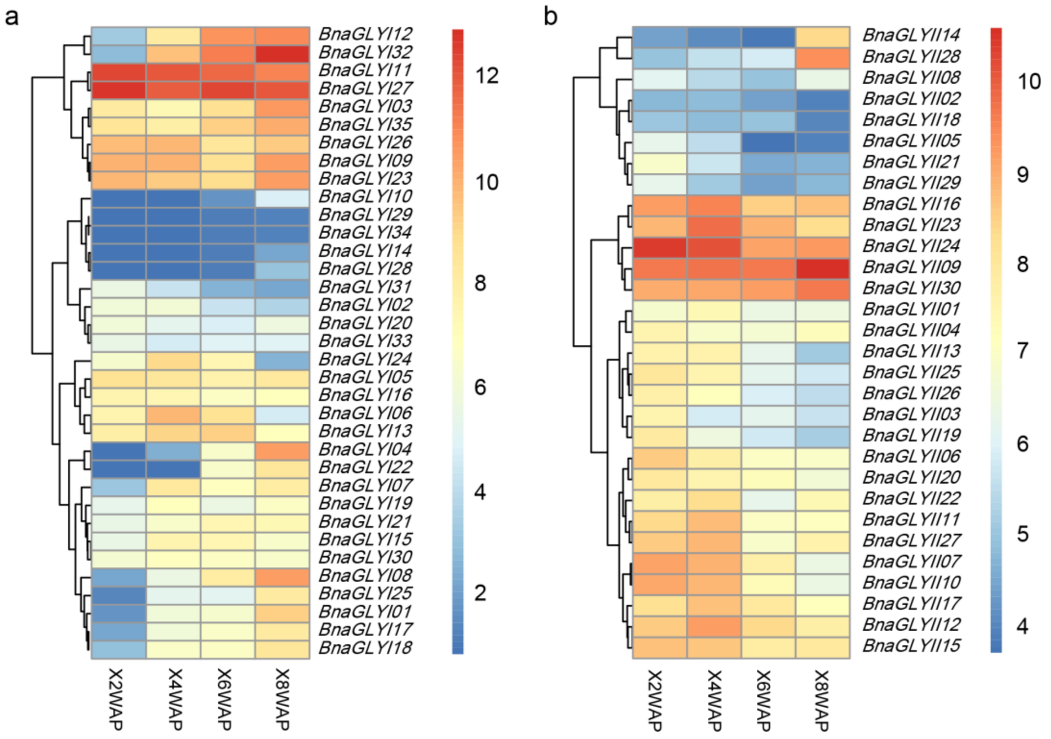

Figure S6. The expression patterns of the *BnaGLY* genes at different seed development times.

a: The expressions of the *BnaGLYI* genes, b： The expressions of the *BnaGLYII* genes

The expression levels of *BnaGLY* in developing seeds at two, four, six and eight weeks after pollination (WAP) were analyzed using the gene expression omnibus (GEO) database (GSE77637)

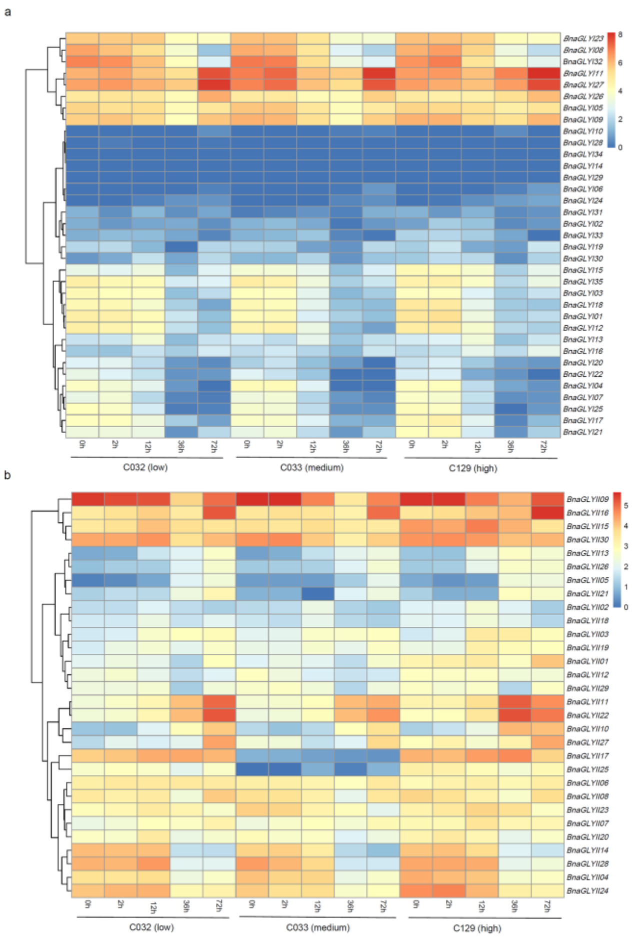

Figure S7. The expressions profiles of the *BnaGLY* genes during seed germination.

a: The expressions of the *BnaGLYI* genes, b：The expressions of the *BnaGLYII* genes

The expression level of *GLY* genes in different seed germination period (0h, 2h, 12h, 36h and 72h) of *B. napus* with different germination rates (high, medium and low; C129, C033 and C032, respectively) using the data in GEO (GSE13723).

Table S1 | Ka and Ks values for *BnaGLYI* paralogous gene pairs

No	Locus 1	Locus 2	Ks	Ka	Ka/Ks	Selection pressure	Average Ka/Ks
1	BnaGLYI11	BnaGLYI27	0.0482	0.0514	1.0664	Positive selection	
2	BnaGLYI14	BnaGLYI29	0.0977	0.0384	0.3930	Purifying selection	
3	BnaGLYI30	BnaGLYI15	0.036	0.1499	4.1639	Positive selection	
4	BnaGLYI30	BnaGLYI21	0.0362	0.1173	3.2403	Positive selection	
5	BnaGLYI32	BnaGLYI04	0.0744	0.1663	2.2352	Positive selection	
6	BnaGLYI32	BnaGLYI22	0.0728	0.1182	1.6236	Positive selection	
7	BnaGLYI03	BnaGLYI35	0.079	0.0134	0.1696	Purifying selection	
8	BnaGLYI33	BnaGLYI05	0.1198	0.2261	1.8873	Positive selection	
9	BnaGLYI33	BnaGLYI23	0.1184	0.2442	2.0625	Purifying selection	1.8559
10	BnaGLYI19	BnaGLYI02	0.0418	0.013	0.3110	Purifying selection	
11	BnaGLYI07	BnaGLYI10	0.2567	0.1386	0.5399	Purifying selection	
12	BnaGLYI07	BnaGLYI28	0.2567	0.1236	0.48150	Purifying selection	
13	BnaGLYI25	BnaGLYI10	0.3206	0.1386	0.4323	Purifying selection	
14	BnaGLYI25	BnaGLYI28	1.3206	0.1236	0.0936	Purifying selection	
15	BnaGLYI23	BnaGLYI05	0.0928	0.066	0.7112	Purifying selection	
16	BnaGLYI22	BnaGLYI12	0.0364	0.2007	5.5137	Positive selection	
17	BnaGLYI10	BnaGLYI28	0.0448	0.0128	0.2857	Purifying selection	
18	BnaGLYI13	BnaGLYI15	0.0358	0.1339	3.7402	Positive selection	
19	BnaGLYI12	BnaGLYI04	0.0372	0.2348	6.3118	Positive selection	

Table S2 | Ka and Ks values for *BnaGLYII* paralogous gene pairs

No	Locus 1	Locus 2	Ks	Ka	Ka/Ks	Selection pressure	Average Ka/Ks
1	BnaGLYII09	BnaGLYII30	0.12470	0.05000	0.4010	Purifying selection	
2	BnaGLYII24	BnaGLYII04	0.05800	0.06350	1.0948	Positive selection	
3	BnaGLYII24	BnaGLYII07	0.02070	0.02720	1.3140	Positive selection	
4	BnaGLYII29	BnaGLYII03	0.11980	0.08840	0.7379	Purifying selection	
5	BnaGLYII29	BnaGLYII08	0.03070	0.03670	1.1954	Positive selection	
6	BnaGLYII25	BnaGLYII06	0.11250	0.03930	0.3493	Purifying selection	
7	BnaGLYII19	BnaGLYII08	0.12060	0.07830	0.6493	Purifying selection	
8	BnaGLYII19	BnaGLYII03	0.04170	0.02420	0.5803	Purifying selection	
9	BnaGLYII10	BnaGLYII27	0.08610	0.03340	0.3879	Purifying selection	
10	BnaGLYII10	BnaGLYII12	0.19390	0.10200	0.5260	Purifying selection	
11	BnaGLYII02	BnaGLYII18	0.01030	0.00890	0.8641	Purifying selection	
12	BnaGLYII28	BnaGLYII14	0.07520	0.05860	0.7793	Purifying selection	0.7978
13	BnaGLYII23	BnaGLYII12	0.05060	0.03380	0.6680	Purifying selection	
14	BnaGLYII08	BnaGLYII03	0.13160	0.07190	0.5464	Purifying selection	
15	BnaGLYII11	BnaGLYII22	0.04280	0.03320	0.7757	Purifying selection	
16	BnaGLYII15	BnaGLYII17	0.05250	0.01200	0.2286	Purifying selection	
17	BnaGLYII13	BnaGLYII26	0.06540	0.04560	0.6973	Purifying selection	
18	BnaGLYII20	BnaGLYII04	0.01020	0.03350	3.2843	Positive selection	
19	BnaGLYII20	BnaGLYII24	0.06890	0.07010	1.0174	Positive selection	
20	BnaGLYII27	BnaGLYII12	0.18670	0.08020	0.4296	Purifying selection	
21	BnaGLYII01	BnaGLYII16	0.06060	0.04020	0.6634	Purifying selection	
22	BnaGLYII21	BnaGLYII05	0.03470	0.00580	0.1672	Purifying selection	
23	BnaGLYII07	BnaGLYII04	0.08040	0.07980	0.9925	Purifying selection	

Table S3 | Analysis of the putative BnaGLYI for their activity and metal ion dependency

Proteins	Metal binding sites Expected GLYI				Expected GLYI enzyme activity
	H/Q	E	H/Q	E	
BnaGLYI1	√	√	√	√	Yes
BnaGLYI2	√	—	√	√	No
BnaGLYI3	√	—	√	√	No
BnaGLYI4	—	√	—	—	No
BnaGLYI5	√	√	√	√	Yes
BnaGLYI6	√	√	√	√	Yes
BnaGLYI7	√	√	√	√	Yes
BnaGLYI8	√	—	√	√	No
BnaGLYI9	√	√	√	√	Yes
BnaGLYI10	√	√	√	√	Yes
BnaGLYI11	√	√	√	√	Yes
BnaGLYI12	—	√	—	—	No
BnaGLYI13	√	—	—	—	No
BnaGLYI14	√	√	√	√	Yes
BnaGLYI15	√	—	—	—	No
BnaGLYI16	√	—	√	√	No
BnaGLYI17	√	√	√	√	Yes
BnaGLYI18	√	√	√	√	Yes
BnaGLYI19	√	—	√	√	No
BnaGLYI20	—	√	—	√	No
BnaGLYI21	√	—	—	—	No
BnaGLYI22	—	√	—	—	No
BnaGLYI23	√	√	√	√	Yes
BnaGLYI24	√	—	√	√	No
BnaGLYI25	√	√	√	√	Yes
BnaGLYI26	√	√	√	√	Yes
BnaGLYI27	√	√	√	√	Yes
BnaGLYI28	√	√	√	√	Yes
BnaGLYI29	√	√	√	√	Yes
BnaGLYI30	√	—	—	—	No
BnaGLYI31	√	—	√	—	No
BnaGLYI32	—	√	—	—	No
BnaGLYI33	—	√	√	√	No
BnaGLYI34	—	—	—	—	No
BnaGLYI35	√	—	√	√	No

Table S4 | Analyses of the putative BnaGLYII proteins for their activity and conserved motifs

Proteins	Conserved metal binding motif (THHHXDH)	Active site motif (C/GHT)	Proteins Expected GLYII enzyme activity
BnaGLYII1	—	√	No
BnaGLYII2	—	—	No
BnaGLYII3	√	√	Yes
BnaGLYII4	√	√	Yes
BnaGLYII5	—	—	No
BnaGLYII6	√	—	No
BnaGLYII7	√	√	Yes
BnaGLYII8	√	√	Yes
BnaGLYII9	—	√	No
BnaGLYII10	—	√	No
BnaGLYII11	—	—	No
BnaGLYII12	—	√	No
BnaGLYII13	√	√	Yes
BnaGLYII14	√	√	Yes
BnaGLYII15	—	—	No
BnaGLYII16	—	√	No
BnaGLYII17	—	—	No
BnaGLYII18	—	—	No
BnaGLYII19	√	√	Yes
BnaGLYII20	√	√	Yes
BnaGLYII21	—	—	No
BnaGLYII22	—	—	No
BnaGLYII23	—	√	No
BnaGLYII24	√	√	Yes
BnaGLYII25	√	√	Yes
BnaGLYII26	√	√	Yes
BnaGLYII27	—	√	No
BnaGLYII28	√	√	Yes
BnaGLYII29	√	√	Yes
BnaGLYII30	√	√	Yes

Table S5. The DNA sequences of *BnaGLY* genes in *B. napus.*

Table S6. The coding sequences of *BnaGLY* genes in *B. napus.*

Table S7. The amino acid sequences of *BnaGLY* genes in *B. napus*.

Table S8.The CT values of the genes (*BnaGLYI29BnaGLYII28* and their corresponding *ACTIN*)

Table S9. The expression level of *BnaGLYI* and *BnaGLYII* family genes under heat and drought stresses.

Table S10. The expression level of *BnaGLYI* and *BnaGLYII* family genes under low temperature stresses.

Table S11. Percentage of similarities among all GLYI proteins used in the phylogenetic analysis

Table S12. Pairwise similarities among GLYII proteins used in the phylogenetic analysis
